# Establishment and validation of the prediction model based on lymphocyte subsets for acute kidney injury in sepsis patients

**DOI:** 10.3389/fimmu.2025.1674673

**Published:** 2025-09-25

**Authors:** Leyi Wang, Qi Liu, Changdong Wu, Ming Hou, Zhiwei Li

**Affiliations:** ^1^ Department of Nursing, People’s Hospital of Xinjiang Uygur Autonomous Region, Urumchi, Xinjiang Uygur Autonomous Region, China; ^2^ Department of Ophthalmology, People’s Hospital of Xinjiang Uygur Autonomous Region, Urumchi, Xinjiang Uygur Autonomous Region, China; ^3^ Xinjiang Emergency Center, People’s Hospital of Xinjiang Uygur Autonomous Region, Urumchi, Xinjiang Uygur Autonomous Region, China; ^4^ Clinical Laboratory Center, People’s Hospital of Xinjiang Uygur Autonomous Region, Urumchi, Xinjiang Uygur Autonomous Region, China

**Keywords:** acute kidney injury, sepsis, lymphocyte subsets, prediction model, nomogram

## Abstract

**Purpose:**

This study aimed to construct a risk predictive model for acute kidney injury in sepsis based on peripheral blood lymphocyte subsets.

**Methods:**

This prospective study included patients with sepsis admitted to the ICU from March to August 2024 (483 for training and 146 for validation), and 125 patients from September to December 2024 as the external test cohort. Clinical data and peripheral blood samples on days 1 and 3 were collected after ICU admission. Lymphocyte subsets were analyzed using flow cytometry, covering T cell, B cell, NK cell populations. Differences in clinical variables and lymphocyte subsets between AKI and non-AKI groups were analyzed. A predictive model was developed using LASSO and multivariate logistic regression and validated internally (5-fold cross-validation) and externally. Model performance was assessed using ROC curves, calibration plots, and decision curve analysis (DCA). A nomogram was constructed for clinical applications.

**Results:**

Among the 483 patients, the incidence of AKI was 54.66%. Compared to non-AKI patients, the AKI group had significantly higher SOFA and APACHE II scores and lower GCS scores. Laboratory findings showed higher neutrophil and monocyte counts, and elevated serum creatinine in the AKI group. On day 1, several lymphocyte subsets were significantly altered in the AKI group, including increased CD4^+^CD38^+^T%, CD8^+^CD38^+^T%, CD155^+^T%, CD4^+^TeM^+^T%, CD8^+^TIGIT^+^T%, and M-MDSC, and decreased CD4^+^LAG3^+^T%, CD4^+^TN^+^T%, and Th17 cells. On day 3, AKI patients exhibited further distinct changes in NK cells and T cell activation/exhaustion markers. A predictive model incorporating key clinical (APACHE II and creatinine) and lymphocyte subsets (CD15^+^T%_1^st^, CD4^+^LAG3^+^T%_1^st^, Th17_1^st^, CD8^+^PD1^+^T%_3^rd^, CD8^+^TIGIT^+^T%_3^rd^, E_MDSC_3^rd^, CD8^+^CCR7^+^CD45RA^+^T%_3^rd^, CD4^+^CTLA4^+^T%_3^rd^, CD4^+^TIM3^+^T%_3^rd^, PMN_MDSC_3^rd^, and M_MDSC_3^rd^) achieved high accuracy, with an AUC of 0.989 in the training set, 0.895 in the validation set, and 0.906 in the test set. Calibration curves and DCA confirmed the model’s reliability and clinical utility.

**Conclusion:**

Peripheral blood lymphocyte subsets are significantly altered in patients who develop SA-AKI and can serve as potential early biomarkers. The developed predictive model based on clinical and immunological parameters demonstrated robust performance in identifying patients at high risk of SA-AKI, offering a practical tool for early warning and clinical decision-making.

## Introduction

Sepsis is a life-threatening condition characterized by organ dysfunction resulting from a dysregulated host response to infection, and it remains a leading cause of mortality in intensive care units (ICUs) (1). According to the Global Burden of Disease Study, approximately 49 million cases of sepsis occur globally each year, with an estimated 11 million sepsis-related deaths. Among critically ill patients, the mortality rate associated with severe sepsis can reach as high as 30–50% (2). Due to its high incidence and mortality, sepsis imposes a substantial economic burden on patients, families, and healthcare systems.

Sepsis is not merely a systemic inflammatory or immune dysregulation process; it is frequently accompanied by multi-organ dysfunction. Among its complications, acute kidney injury (AKI) is one of the most common and severe. When AKI occurs as a consequence of sepsis, it is referred to as sepsis-associated acute kidney injury (SA-AKI). SA-AKI is defined by a rapid decline in renal function, leading to the accumulation of nitrogenous waste products and disturbances in electrolyte and acid-base homeostasis (3). Compared to AKI resulting from other causes, SA-AKI is associated with significantly prolonged hospital stays and increased in-hospital mortality (4). Notably, the mortality rate in septic patients with concurrent AKI may be as high as 70% (5). Despite advances in supportive care and diagnostic technologies, the prognosis of SA-AKI remains poor, and its diagnosis still relies on conventional indicators such as changes in serum creatinine and urine output. These markers lack sensitivity and specificity for detecting early pathophysiological changes (6). The underlying mechanisms of SA-AKI are complex and heterogeneous, involving inflammation, complement activation, dysregulation of the renin–angiotensin–aldosterone system (RAAS), mitochondrial dysfunction, microcirculatory disturbances, and metabolic reprogramming (7). Over recent decades, various biomarkers have been explored for the early prediction of AKI, including neutrophil gelatinase-associated lipocalin (NGAL), kidney injury molecule-1 (KIM-1), tissue inhibitor of metalloproteinase-2 (TIMP-2), and insulin-like growth factor-binding protein 7 (IGFBP7), and C-reactive protein-albumin-lymphocyte index (8–11). However, many of these biomarkers are limited in clinical utility due to high cost, technical complexity, and lack of accessibility. Therefore, there is an urgent need for simple, reliable, and widely available biomarkers for the early identification and risk stratification of SA-AKI.

Lymphocyte subsets, a group of white blood cells, are essential components of the immune system and play pivotal roles in immune regulation. During immune responses, lymphocytes differentiate into functional subtypes, including T lymphocytes, B lymphocytes, and natural killer (NK) cells (12). Flow cytometry is routinely used in clinical practice to assess immune status by quantifying these subsets. T cells are primarily responsible for eliminating infected and malignant cells; B cells generate antibodies for antigen-specific responses; and NK cells target virus-infected cells by recognizing stress ligands (12). Alterations in lymphocyte subset counts have been associated with the severity and prognosis of a variety of diseases, including sepsis (13–15). Accumulating evidence suggests that the onset and progression of sepsis are closely tied to immune dysfunction, particularly immunosuppression mediated by decreased T cell reactivity (16, 17). Sepsis-induced immune dysregulation, often underpinned by systemic inflammatory response syndrome (SIRS), is characterized by widespread lymphocyte apoptosis and functional exhaustion. T cell apoptosis contributes significantly to immune paralysis and T cell clonal anergy (18, 19). Among T cell subsets, CD3^+^ T cells reflect overall cellular immunity, CD4^+^ T cells (helper T cells) assist in coordinating the immune response, and CD8^+^ T cells (cytotoxic/suppressor T cells) are involved in immune inhibition (20). Thus, alterations in the absolute counts and ratios of CD3^+^, CD4^+^, CD8^+^ T cells, and the CD4^+^/CD8^+^ ratio can reflect changes in cellular immune status (21). Recent clinical studies have reported that septic patients with AKI exhibit distinct alterations in CD4^+^ T cell subsets (22), and that sepsis-induced AKI is often accompanied by T lymphopenia (23). Animal studies have further shown that peripheral T cell apoptosis is mechanistically linked to the development of SA-AKI, providing a theoretical foundation for further investigation (24). Given the clinical availability and simplicity of lymphocyte subset testing, these parameters represent a promising avenue for non-invasive, early detection of SA-AKI. However, their predictive value remains underexplored in this context.

In this study, we performed a comprehensive analysis of peripheral blood lymphocyte subsets including T cells, B cells, and NK cells in patients with sepsis, using flow cytometry. By integrating lymphocyte profiles with clinical features, we constructed a predictive model for SA-AKI occurrence. The model underwent both internal and external validation, culminating in an individualized risk assessment tool for SA-AKI. This work aims to provide a practical framework for early warning, improved clinical decision-making, and optimized management of patients with sepsis-associated acute kidney injury.

## Material and methods

### Study population

This study was designed as a prospective investigation. Baseline data and blood samples were collected from 767 sepsis patients admitted to the intensive care unit (ICU) of our hospital between March 7, 2024, and August 30, 2024. To reduce the potential impact of confounding factors, predefined inclusion and exclusion criteria were applied to identify eligible participants. Sepsis was diagnosed according to the Sepsis-3 criteria (25). The inclusion criteria were as follows: (1) Diagnosis of sepsis based on Sepsis-3, defined as an increase of ≥2 points in the Sequential Organ Failure Assessment (SOFA) score from baseline in patients with confirmed or suspected infection; (2) Age >18 years.

**Figure 1:**
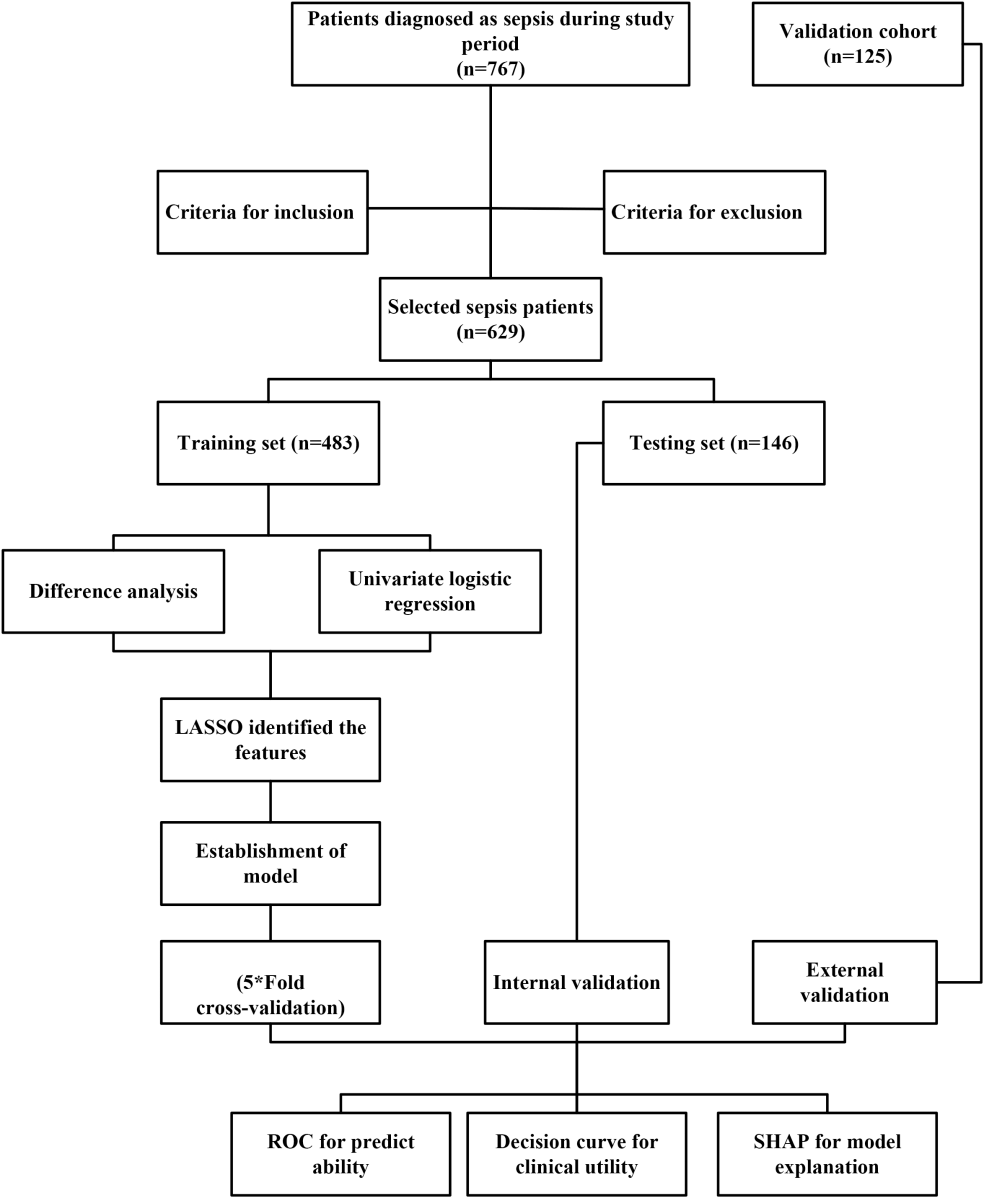
Flow chart of the whole study.

(3) ICU stays longer than 24 hours. The exclusion criteria were: (1) Age <18 years; (2) ICU stay <24 hours or development of AKI within 36 hours of admission; (3) History of kidney disease, dialysis, cancer, hematologic or immune system disorders, or severe liver dysfunction; (4) Presence of AKI before ICU admission; (5) Missing baseline data or loss to follow-up, or missing >10% of laboratory data. Based on these inclusion and exclusion criteria, 629 patients with sepsis were assigned to the training set (n=483) and validation set (n=146). For binary logistic regression, the empirical rule of thumb requires the sample size (N) to be 10–20 times the number of independent variables (k) *N*≥10–20×*k*. In this study, 23 independent variables were included in the initial screening (k = 23). Therefore, the minimum required sample size was estimated as: N_min_=10×23×(1 + 10%)=253. where 10% was added to account for potential loss to follow-up. Finally, 629 participants were included in the analysis, which substantially exceeds the minimum requirement, ensuring adequate statistical power. An external test cohort consisting of 125 additional sepsis patients from the same institution was identified between September 1, 2024, and December 31, 2024. The follow-up period for all patients was one month. **Figure 1** presented the study process.

### Data collection and definitions

The data collection included clinical information and blood samples. The clinical information consisted of age (year), gender (male vs female), body mass index (BMI=weight(kg)/height(m)^2^), SOFA score, acute physiology and chronic health evaluation II (APACHEII), and Glasgow Coma scale (GCS). The history of diseases: chronic obstructive pulmonary disease (COPD), hypertension (Systolic blood pressure≥140mm Hg and/or diastolic blood pressure≥90mm Hg), diabetes (Fasting plasma glucose≥7.0 mmol/L or Oral glucose tolerance test≥11.1 mmol/L), coronary heart disease (CHD), primary infection, septic shock, (Yes vs No), continuous renal replacement therapy (CRRT) and vasoactive drugs usage. The blood samples were collected on the first day and the third day after admission. The biochemical parameters were analyzed, including white blood cells (WBC, 10^9^), hemoglobin (g/L), platelet (10^9^), neutrophil (10^9^), lymphocyte (10^9^), monocyte (10^9^), C-reactive protein (mg/L), procalcitonin (ng/L), total bilirubin (μmol/L), albumin (g/L), globulin (g/L), blood urea nitrogen (BUN, mmol/L), creatinine (μmol/L), and lactate (mmol/L).

The peripheral blood lymphocyte subsets of patients with sepsis on the first and third days were detected by flow cytometry. To comprehensively evaluate the role of lymphocyte subgroups in the development of AKI among patients with sepsis, we employed established detection methods to systematically measure the relevant indicators within each lymphocyte subset. The lymphocyte subsets included T cell subsets (CD3+T%, CD4+T%, CD8+T%, CD4+CD8+T%, CD4-CD8-T%, CD3+T count, CD4+T count, CD8+T count, CD4/CD8, CD4+CD8+T count, CD4-CD8-T count, CD4+CD28+T%, CD4+CD38+T%, CD4+CD69+T%, CD8+CD28+T%, CD8+CD38+T%, CD8+CD69+T%, CD155+T%, CD4+BTLA+T%, CD4+CTLA4+T%,CD4+HLADR+T%, CD4+LAG3+T%,CD4+PD1+T%,CD4+TIGIT+T%, CD4+TIM3+T%, CD4+TcM+T%, CD4+TeM+T%, CD4+TeMRA+T%, CD4+TN+T%, CD8+BTLA+T%,CD8+CTLA4+T%,CD8+HLADR+T%,CD8+LAG3+T%,CD8+PD1+T%,CD8+TIGIT+T%,CD8+TIM3+T%, CD8+TcM+T%,CD8+TeM+T%, CD8+TeMRA+T%, CD8+TN+T%, MDSC, PMN_MDSC, M_MDSC, e_MDSC, Th1, Th2, Th17, Treg, CD4+CD45RA+T%, CD4+CD45RO+T%, CD8+CD45RA+T%, CD8+CD45RA+T%, CD4+CCR7+CD45+T%, CD4+CCR7+CD45-T%, CD4+CCR7-CD45+T%, CD4+CCR7-CD45-T%, CD8+CCR7+CD45+T%, CD8+CCR7+CD45-T%, CD8+CCR7-CD45+T%, and CD8+CCR7-CD45-T%), B cells subsets (CD19+B count), lymphocyte count, NK cells subsets (NKT count, CD16+CD56+NK%, CD16+CD56+NK count, NKT%), and B cells subsets (CD19+B%), and Neutrophil CD64 index (nCD64).

According to the diagnostic criteria of Kidney Disease Improving Global Outcomes (KDIGO) (26), the acute kidney injury (AKI) is defined as follows: Serum creatinine increase of ≥0.3 mg/dL (within 48 hours) and/or ≥50% increase from baseline creatinine (within 7 days).

### Statistical analysis

All data analyses were performed using R version 4.4.3. The online tool Sangerbox 2 was also used to plot the forest plot (27). For continuous variables with a normal distribution (age, BMI, SOFA, APACHEII, GCS, WBC, hemoglobin, platelet, neutrophil, lymphocyte, monocyte, C-reactive protein, procalcitonin, total bilirubin, albumin, globulin, BUN, creatinine, and lactate), data are presented as mean ± standard deviation, and comparisons between two groups were conducted using the independent samples t-test. For non-normally distributed continuous variables (lymphocyte subsets), data are presented as median and interquartile range, and comparisons between groups were performed using the Wilcoxon test. The false discovery rate was applied for multiple comparisons of lymphocyte subsets. Categorical variables are presented as counts and percentages (gender, COPD, hypertension, diabetes, CHD, primary infection, septic shock, CRRT, and vasoactive drugs usage), and comparisons between groups were conducted using the Chi-square test. *P* < 0.05 was considered a significant level.

To develop a predictive model for AKI in patients with sepsis, we used the training set to identify overlapping variables between those showing significant differences between the AKI and non-AKI groups and those identified as significant in univariate logistic regression. These overlapping variables were then further refined using the least absolute shrinkage and selection operator (LASSO) regression. Subsequently, multivariate logistic regression with forward stepwise selection was performed. Variables with a p-value < 0.05 were retained in the final predictive model.

The model was validated using two approaches. For internal validation, five random subsets were extracted from the training set and subjected to 5-fold cross-validation. For external validation, an independent cohort dataset was used. Model performance was assessed using the receiver operating characteristic (ROC) curve to evaluate discriminative ability. Calibration plots for both the training and validation sets were generated to assess the agreement between predicted and observed probabilities, demonstrating the model’s stability.

A nomogram for predicting the risk of AKI was constructed using the R “rms” package based on the multivariate logistic regression model. Finally, decision curve analysis was conducted to evaluate the clinical utility of the model by weighing its potential benefits and risks, thereby determining its applicability in clinical decision-making. The SHapley Additive explanation (SHAP) method was used for global and local explanations for the model explanation.

## Results

### Clinical characteristics between non-AKI and AKI groups

Based on the predefined inclusion and exclusion criteria, a total of 483 patients with sepsis were included in the training set. The incidence of acute kidney injury (AKI) was 54.66% (264/483). **Table 1** summarizes the comparisons of clinical characteristics between the AKI and non-AKI groups. No significant differences were observed in age (*P* = 0.971), gender distribution (*P* = 0.286), or body mass index (BMI) (*P* = 0.509) between the two groups. However, the SOFA score (8.94 ± 3.67 vs. 6.89 ± 3.48, *P* < 0.001) and the APACHE II score (22.86 ± 6.93 vs. 17.00 ± 7.87, *P* < 0.001) were significantly higher in the AKI group compared to the non-AKI group. In contrast, the GCS score was significantly lower in the AKI group (10.16 ± 3.60 vs. 11.52 ± 3.76, *P* = 0.020). There were no significant differences in the prevalence of hypertension (*P* = 0.214), diabetes mellitus (*P* = 0.573), or coronary heart disease (CHD) (*P* = 0.702) between the groups. However, the prevalence of chronic obstructive pulmonary disease (COPD) (11.36% vs. 0.00%, *P* = 0.008), use of continuous renal replacement therapy (CRRT) (39.77% vs. 20.55%, *P* = 0.009), administration of vasoactive drugs (77.27% vs. 56.16%, *P* = 0.004), occurrence of septic shock (80.68% vs. 54.79%, *P* < 0.001), and rate of primary infection (94.32% vs. 83.56%, P = 0.027) were all significantly higher in the AKI group.

**Table 1:** Comparisons of clinical characteristics between non-AKI and AKI groups.

Variables	Non-AKI	AKI	*P*
Age, year	66.07 ± 17.21	65.98 ± 13.94	0.950
Gender, Male (%)	90 (41.10)	87 (32.95)	0.065
BMI, kg/m^2^	24.82 ± 3.52	25.24 ± 4.57	0.251
SOFA	6.89 ± 3.47	8.94 ± 3.66	<0.001
APACHEII	17.00 ± 7.84	22.86 ± 6.90	<0.001
GCS	11.52 ± 3.74	10.16 ± 3.59	<0.001
COPD, n (%)	0 (0.00)	30 (11.36)	<0.001
Hypertension, n (%)	93 (42.47)	87 (32.95)	0.031
Diabetes, n (%)	66 (30.14)	69 (26.14)	0.329
CHD, n (%)	66 (30.14)	87 (32.95)	0.508
CRRT, n (%)	45 (20.55)	105 (39.77)	<0.001
Vasoactive drugs, n (%)	123 (56.16)	204 (77.27)	<0.001
Septic shock, n (%)	40 (54.79)	71 (80.68)	<0.001
Primary Infection, n (%)	183 (83.56)	249 (94.32)	<0.001
Whtie blood cell, 10^9^/L	12.90 ± 7.09	15.03 ± 8.76	0.003
Hemoglobin, g/L	113.37 ± 31.48	107.86 ± 29.67	0.049
Platelet, 10^9^/L	171.53 ± 92.37	164.33 ± 113.35	0.442
Neutrophil, 10^9^/L	12.41 ± 11.33	20.77 ± 30.45	<0.001
Lymphocyte, 10^9^/L	0.90 ± 0.84	1.68 ± 3.32	<0.001
Monocyte, 10^9^/L	0.53 ± 0.40	0.84 ± 0.91	<0.001
C-reactive protein, mg/L	134.95 ± 84.79	138.76 ± 104.18	0.658
Procalcitonin, ng/L	20.51 ± 30.80	28.11 ± 33.66	0.010
Total bilirubin, μmol/L	42.87 ± 48.67	38.48 ± 41.95	0.288
Albumin, g/L	30.57 ± 6.62	29.65 ± 5.75	0.102
Globulin, g/L	26.79 ± 4.68	27.09 ± 5.22	0.515
Blood urea nitrogen, mmol/L	14.51 ± 9.10	17.94 ± 12.36	<0.001
Creatinine, μmol/L	128.09 ± 131.74	261.19 ± 258.47	<0.001
Lactate, mmol/L	3.06 ± 2.75	5.33 ± 18.43	0.071

In terms of laboratory findings, both neutrophil count (20.77 ± 30.56 vs. 12.41 ± 11.38, *P* = 0.019) and monocyte count (0.84 ± 0.91 vs. 0.53 ± 0.40, *P* = 0.005) were significantly elevated in the AKI group. Serum creatinine levels were also markedly higher in the AKI group (261.19 ± 259.45 vs. 128.09 ± 132.35, *P* < 0.001). No significant differences were found between the groups in white blood cell count (*P* = 0.098), hemoglobin level (*P* = 0.258), platelet count (*P* = 0.665), lymphocyte count (*P* = 0.056), C-reactive protein (*P* = 0.803), procalcitonin (*P* = 0.142), total bilirubin (*P* = 0.542), albumin (*P* = 0.348), globulin (*P* = 0.711), blood urea nitrogen (BUN) (*P* = 0.052), or lactate level (*P* = 0.301).

### Lymphocyte subsets between non-AKI and AKI groups

**Figure 2:**
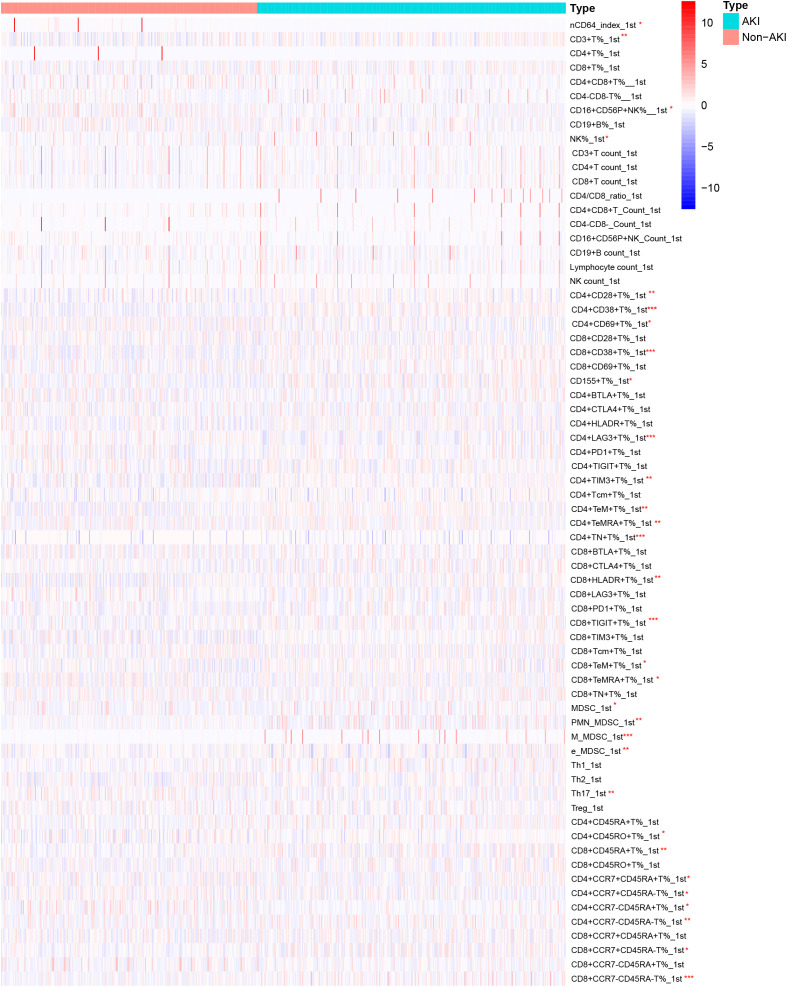
Heatmap of lymphocyte subset levels on the first day for AKI and non-AKI in sepsis patients.

The first day and third day lymphocyte subsets were compared between non-AKI and AKI groups. **Figure 2** shows the heatmap of the lymphocyte subsets on the first day between two groups. Compared with the non-AKI group, the AKI group exhibited elevated levels of CD4+CD38+T% (*P* < 0.011), CD8+CD38+T% (*P* < 0.001), and CD155+T% (*P* = 0.016). Additionally, CD4+TeM+T% (*P* = 0.001), CD8+TIGIT+T% (*P* = 0.012), M-MDSC (*P* < 0.001), and CD8+CCR7-CD45RA-T% (*P* = 0.026) levels were increased in the AKI group. In contrast, CD4+LAG3+T% (*P* = 0.017), CD4+TN+T% (*P* < 0.001), and Th17 (*P* = 0.014) levels were decreased in the AKI group. No significant differences were observed in other lymphocyte subsets between the two groups (*P*>0.005). More detailed information is provided in **Supplementary Table S1**.

**Figure 3:**
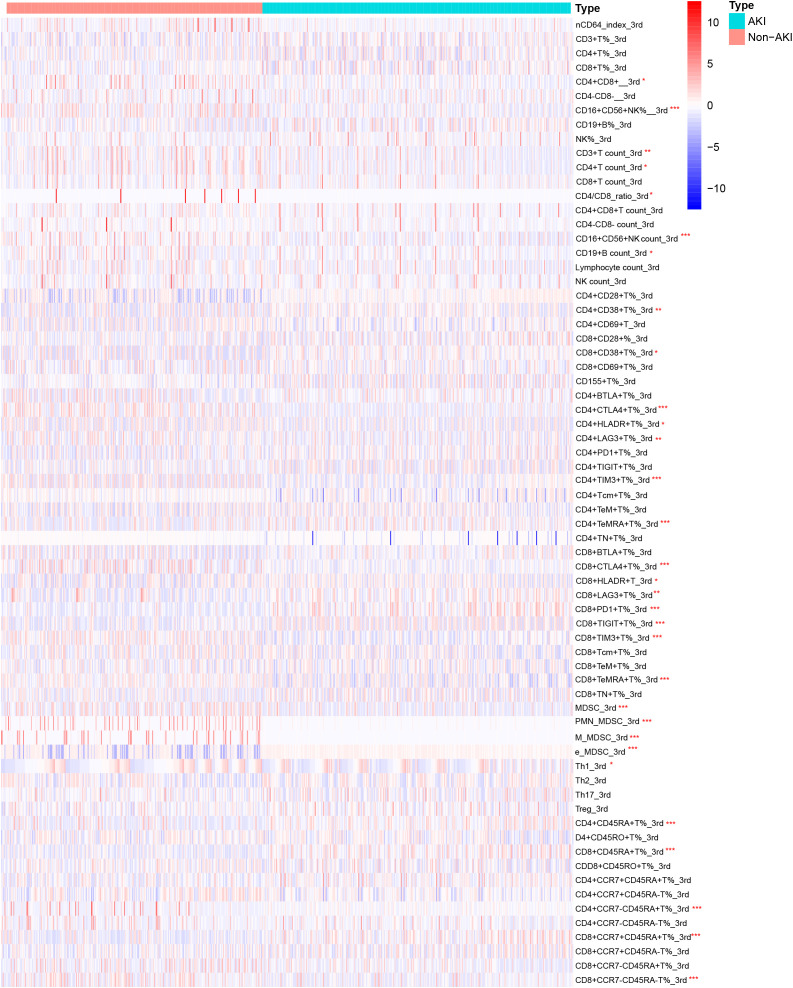
Heatmap of lymphocyte subset levels on the third day for AKI and non-AKI in sepsis patients.

**Figure 3** showed the heatmap of the lymphocyte subsets on the third day between the two groups. Compared with the non-AKI group, the AKI group had lower levels of CD16+CD56+NK% (*P* = 0.036), CD16+CD56+NK count (*P* = 0.005), CD4+CTLA4+T% (*P* < 0.001), CD4+TIM3+T% (*P* = 0.002), CD8+CTLA4+T% (*P* = 0.009), CD8+TIM3+T% (*P* < 0.001), CD8+TeMRA+T% (*P* = 0.038), MDSC (*P* = 0.043), e_MDSC (*P* = 0.017), and CD8+CCR7-CD45-T% (*P* = 0.001). However, the levels of CD4+TeMRA+T% (*P* = 0.043), CD8+PD1+T% (*P* = 0.001), CD8+TIGIT+T% (*P* = 0.005), PMN_MDSC (*P* = 0.002), M_MDSC (*P* = 0.004), CD4+CD45RA+T% (*P* = 0.002), CD4+CCR7-CD45+T% (*P* = 0.008), and CD8+CCR7+CD45+T% (*P* = 0.006) were significantly higher in the AKI group than in the non-AKI group. No significant differences were observed for other lymphocyte subsets, and more detailed information can be found in **Supplementary Table S2**.

### Establishment of the prediction model for AKI in sepsis patients

**Figure 4:**
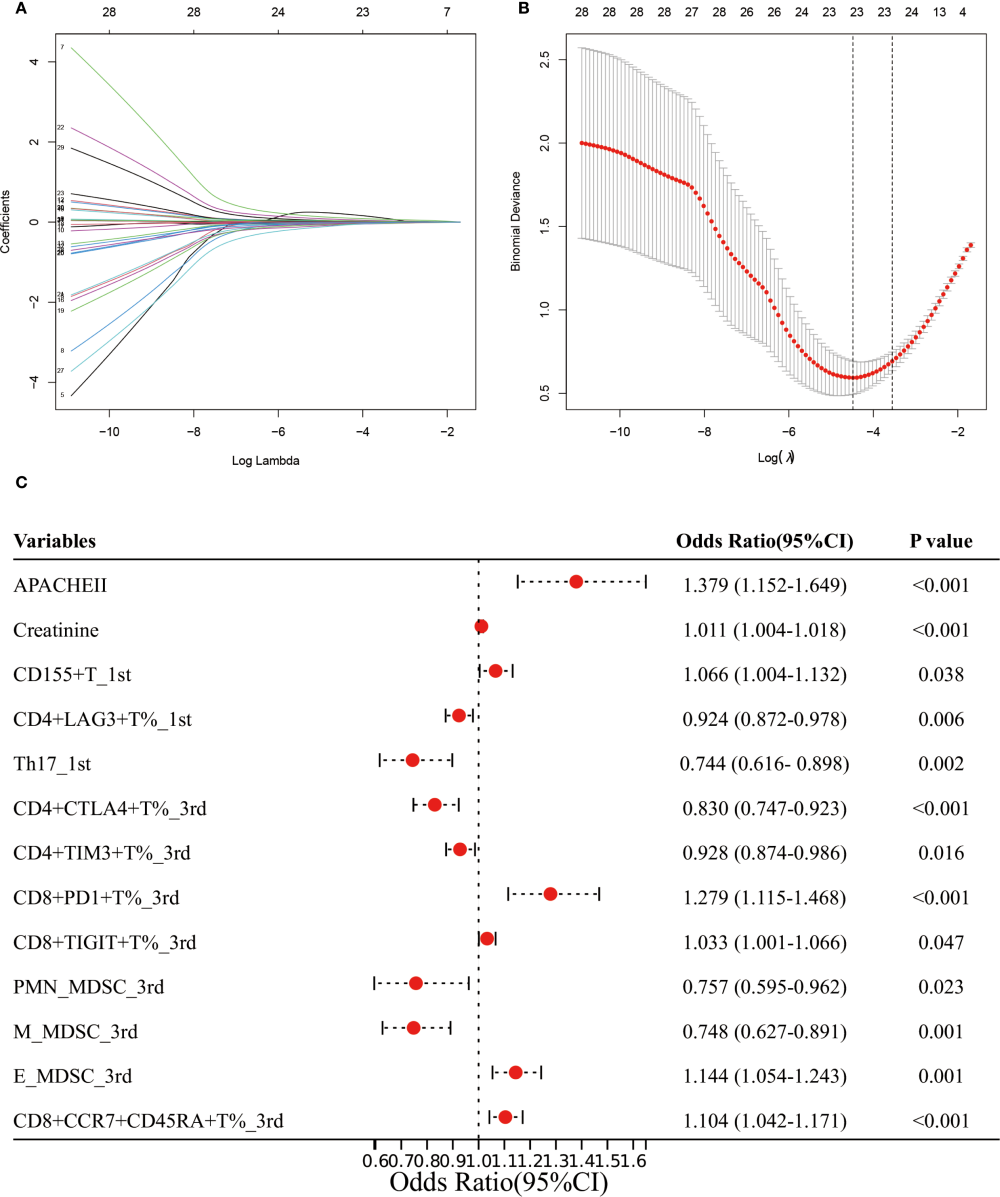
Establishment of the prediction model for AKI in sepsis patients. **(A, B)** LASSO regression identified the potential model factors. **(C)** Forest plot of multivariate logistic regression for AKI in sepsis patients.

We identified 32 factors by overlapping the variables that showed significant differences (**Supplementary Table S1**, **S2**) between the AKI and non-AKI groups with those found to be significant in univariate logistic regression (**Supplementary Table S3**). These 32 factors were then subjected to LASSO regression, resulting in the selection of 23 variables for further analysis (**Figures 4A, B**). A multivariate logistic regression using a forward stepwise approach identified 13 factors associated with the risk of AKI in sepsis patients. Specifically, higher values of APACHE II (OR: 1.379, 95% CI: 1.152-1.649), creatinine (OR: 1.011, 95% CI: 1.004-1.018), CD15^+^T%_1st (OR: 1.066, 95% CI: 1.004-1.132), CD8^+^PD1^+^T%_3rd (OR: 1.279, 95% CI: 1.115-1.468), CD8^+^TIGIT^+^T%_3rd (OR: 1.033, 95% CI: 1.001-1.066), E_MDSC_3rd, and CD8^+^CCR7^+^CD45RA^+^T%_3rd were associated with an increased risk of AKI. In contrast, higher levels of CD4^+^LAG3^+^T%_1st (OR: 0.924, 95% CI: 0.872-0.978), Th17_1st (OR: 0.744, 95% CI: 0.616-0.898), CD4^+^CTLA4^+^T%_3rd (OR: 0.830, 95% CI: 0.747-0.923), CD4^+^TIM3^+^T%_3rd (OR: 0.928, 95% CI: 0.874-0.986), PMN_MDSC_3rd (OR: 0.757, 95% CI: 0.595-0.962), and M_MDSC_3rd (OR: 0.748, 95% CI: 0.627-0.891) were associated with a decreased risk of AKI (**Figure 4C**).

### Validation and assessment of the prediction model for AKI in sepsis patients

**Figure 5:**
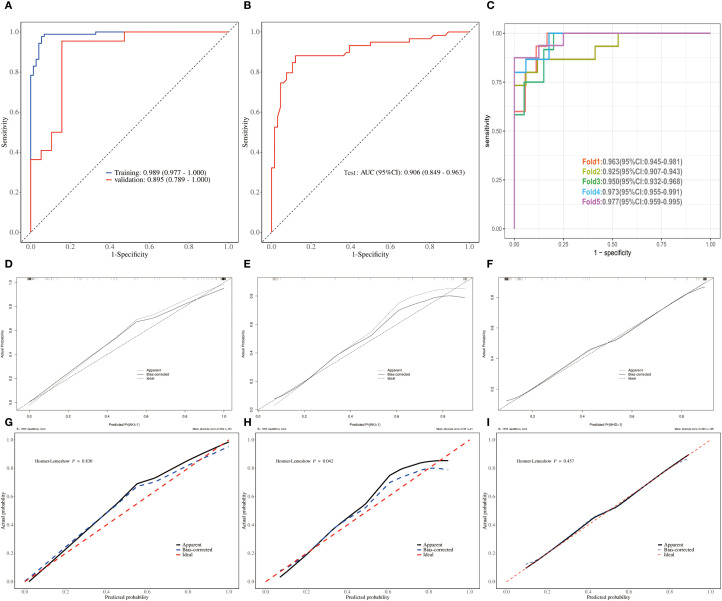
Validation and assessment of the predict model for AKI in sepsis patients. **(A, B)** ROCs of training, validation and test sets. **(C)** ROCs of 5-fold cross-validation. **(D–F)** Crude calibration plots of crude for training, validation and test set. **(G–I)** Corrected calibration plots of crude for training and validation set.

We first compared the clinical characteristics and lymphocyte subsets in the training, validation and test sets and found no significant differences among the three groups. There is good comparability between among the training, the validation and test sets (**Supplementary Tables S4**–**S6**). ROC analysis showed that the AUC was 0.989 (95% CI: 0.977-1.000) in the training set, 0.895 (95% CI: 0.789-1.000) in the validation set (**Figure 5A**), and 0.906(95%CI: 0.849-0.963) in the test set (**Figure 5B**). To further evaluate the model’s robustness, we performed five-fold cross-validation. The AUCs for Fold 1 to Fold 5 were 0.963 (95% CI: 0.945-0.981), 0.925 (95% CI: 0.907-0.943), 0.950 (95% CI: 0.932-0.968), 0.973 (95% CI: 0.955-0.991), and 0.977 (95% CI: 0.959-0.995), respectively (**Figure 5C**).

**Figure 6:**
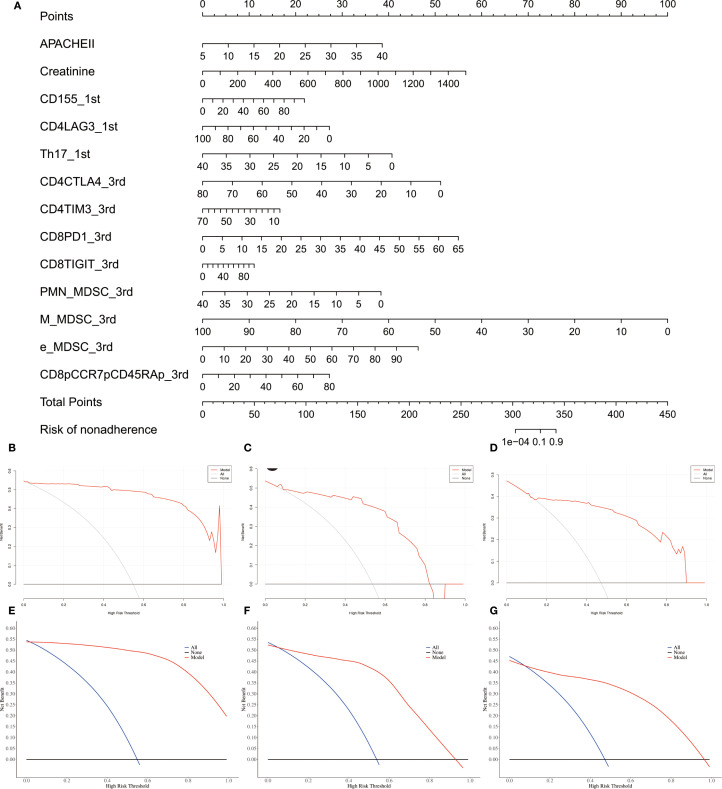
Assessment of individual’s benefits and risk for sepsis patients. **(A)** Nomogram plot for individual risk assessment in sepsis patients. **(B–D)** Crude decision curves of training, validation and test sets. **(E–G)** Adjusted decision curves of training, validation and test.

**Figure 7:**
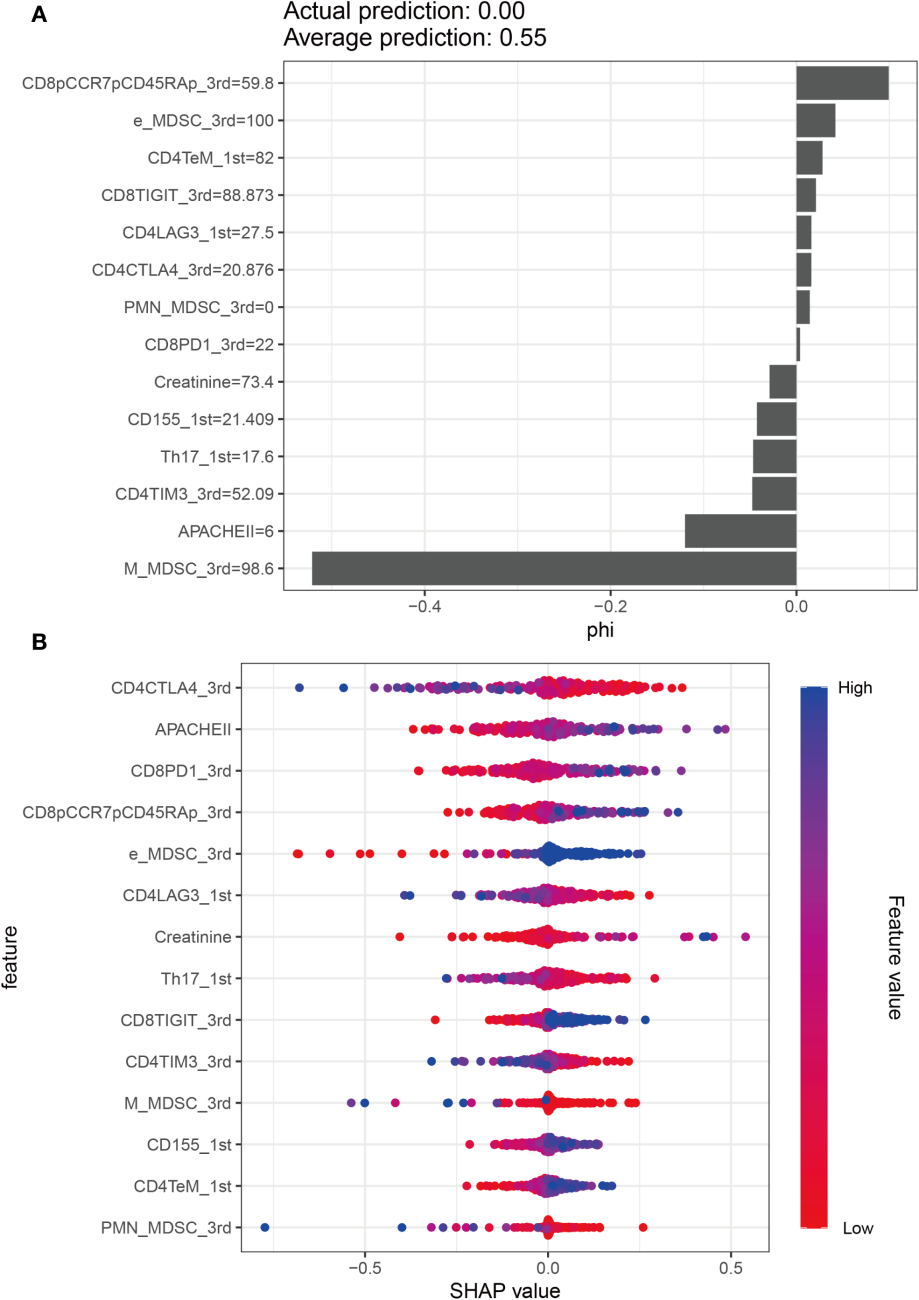
Model explanation by the SHAP method. **(A)** SHAP summary bar plot. **(B)** SHAP summary dot plot.

Unadjusted calibration plots for both the training, validation and test sets demonstrated good agreement between predicted and observed probabilities (**Figures 5D–F**). After adjustment, the calibration plots showed reduced bias and improved consistency (**Figures 5G–I**). Based on the 13 predictors included in the final model, we developed an individualized risk scoring system (**Figure 6A**). Decision curve analysis indicated that patients in the training set could benefit from clinical decisions guided by the model (**Figure 6B**). In the validation and test set, the model provided clinical net benefit when the decision threshold ranged from 0.10 to 0.84 (**Figures 6C, D**). After adjustment, the model continued to show clinical utility, with net benefit observed across a decision threshold range of 10% to 90% (**Figures 6E–G**). As shown in SHAP summary plots (**Figures 7A, B**), the importance of variables was evaluated using the contribution of variables to the model. The larger the absolute SHAP value, the greater the contribution to the model and the more important the feature is. The SHAP dot plot can judge the effect of variables on the AKI. The high feature value can increase the risk of AKI, such as patients with high APACHE II, creatinine, CD155+T_1^st^, CD8+PD1+T%_3^rd^, CD8+TIGIT+T%_3^rd^, E_MDSC_3^rd^, and CD8+CCR7+CD45RA+T% (SHAP value>0), which pushed the decision towards the AKI group. In contrast, patients with the low levels of CD4+LAG3+T%_1^st^, Th17_1^st^, CD4+CTLA4+T%_3^rd^, CD4+TIM3+T%_3^rd^, PMN_MDSC_3^rd^, and M_MDSC_3^rd^ had SHAP values lower than zero, which pushed the decision towards the AKI group.

## Discussion

Sepsis-associated acute kidney injury (SA-AKI) is a severe and life-threatening condition characterized by high mortality and a lack of specific clinical features. The pathophysiology of sepsis is complex and often involves multiple organ systems, complicating both diagnosis and treatment. Due to the unique pathophysiological characteristics of SA-AKI, its clinical management is particularly challenging, with high mortality rates and poor prognosis (28, 29). Sepsis is one of the most common causes of AKI, and conversely, AKI can serve as an early indicator of sepsis. However, elucidating the molecular mechanisms underlying SA-AKI remains extremely difficult (30). Currently, there are no specific or standardized therapeutic strategies for the treatment of SA-AKI, posing significant challenges to early diagnosis and effective intervention (31). These challenges ultimately contribute to poor outcomes in affected patients. Therefore, early assessment and timely treatment of SA-AKI are critical for improving patient prognosis. This study investigated the clinical characteristics and lymphocyte subset changes in sepsis patients with Acute Kidney Injury (AKI), aiming to develop and validate a predictive model for AKI based on these factors. Our study will offer a promising tool for early AKI risk assessment in sepsis patients.

Previous studies reported that the incidence of AKI was approximately 50% in sepsis patients. In a large prospective cohort study spanning 24 European countries and 198 ICUs, 1177 sepsis patients were observed to have a 51% incidence of AKI and a 41% ICU mortality rate (32). A retrospective study in China, encompassing 146,148 sepsis patients, reported an AKI incidence of 47.1% (33). An ancillary analysis of a multicenter RCT on septic shock, encompassing 1,243 patients, found that 50.4% had AKI upon emergency department admission, with a further 18.7% developing AKI within the subsequent seven days (34, 35). The incidence of AKI among septic patients in our cohort was 54.66%, which is consistent with previously reported rates in critically ill populations. We found Some clinical characteristics were significantly different between AKI and non-AKI groups. Compared to the non-AKI group, AKI patients exhibited significantly higher SOFA and APACHE II scores, but lower GCS scores. The SOFA and APACHE II scores were associated with diseases severity and prognosis, and the lower GCS was related to the degree of coma (36, 37). Regarding comorbidities and interventions, the AKI group showed significantly higher prevalence of COPD, CRRT use, vasoactive drug administration, septic shock occurrence, and primary infection rates. Laboratory analyses revealed elevated neutrophil count, monocyte count, and serum creatinine levels in the AKI group. These changes in the clinical characteristics of AKI patients could be associated with reduced bilateral glomerular perfusion, inflammatory responses, metabolic adaptations, and impaired microcirculatory function, which are key mechanisms of organ damage in sepsis patients (38). However, only APACHE II and creatinine were included in the final prediction model for AKI in sepsis. That makes sense, as serum creatinine is a primary indicator for diagnosing sepsis-associated AKI, while the APACHE II score reflects the overall severity of illness in sepsis patients (5, 39).

Although the precise role of lymphocyte subsets in sepsis-induced acute kidney injury (AKI) remains unclear, several studies provide important insights. It has been suggested that damage-associated molecular patterns (DAMPs), such as high mobility group box 1 (HMGB1), cell-free DNA (cfDNA), and histones, can be released as a result of widespread immune cell death. These molecules can trigger endothelial injury and microcirculatory dysfunction, thereby contributing to the progression of multiple organ failure in patients with sepsis (40). In our study, we observed significant alterations in lymphocyte subsets between the AKI and non-AKI groups on both the first- and third days following sepsis onset. On day 1, the AKI group exhibited significantly elevated levels of CD4^+^CD38^+^ T%, CD8^+^CD38^+^ T%, CD15^+^ T%, CD4^+^TeM^+^ T%, CD8^+^TIGIT^+^ T%, monocytic myeloid-derived suppressor cells (M-MDSCs), and CD8^+^CCR7⁻CD45RA⁻ T%. In contrast, levels of CD4^+^LAG3^+^ T%, CD4^+^TN^+^ T%, and Th17 cells were significantly reduced. By day 3, the AKI group showed notably decreased levels of CD16^+^CD56^+^ NK% and NK cell count, CD4^+^CTLA4^+^ T%, CD4^+^TIM3^+^ T%, CD8^+^CTLA4^+^ T%, CD8^+^TIM3^+^ T%, CD8^+^TeMRA^+^ T%, total MDSCs, and early-stage MDSCs (e-MDSCs). Conversely, the same group had significantly increased levels of CD4^+^TeMRA^+^ T%, CD8^+^PD1^+^ T%, CD8^+^TIGIT^+^ T%, polymorphonuclear MDSCs (PMN-MDSCs), M-MDSCs, CD4^+^CD45RA^+^ T%, CD4^+^CCR7⁻CD45^+^ T%, and CD8^+^CCR7^+^CD45^+^ T%. These findings suggest that lymphocyte subset dysfunction is not limited to peripheral blood but may also affect target organs through systemic circulation. Such dysfunction has been associated with the dysregulated release of immunoregulatory molecules, including excessive pro-inflammatory cytokines, which can intensify tissue inflammation and injury (41). For instance, Akcay et al. reported that CD4^+^ T cells contribute to neutrophil recruitment and apoptosis in a murine model of cisplatin-induced AKI. These cells also upregulated IL-33 expression and promoted inflammatory factor release, ultimately causing immune-mediated injury to the peritubular and glomerular capillary networks (42). Mesenchymal stem cells attenuate sepsis-AKI by changing the balance of Th17 cells/Tregs via Gal-9/Tim-3 (43). Conversely, certain interventions have shown promise in mitigating this damage. Curcumin, a well-known anti-inflammatory compound, has been shown to alleviate kidney and lung inflammation by enhancing the suppressive activity of regulatory T cells (Tregs) (44). Similarly, glutamine supplementation can attenuate renal injury by promoting balanced T cell polarization and reducing T cell apoptosis (44). Immune regulation is further modulated by immune checkpoint molecules expressed on lymphocytes and antigen-presenting cells, such as PD-1 and PD-L1, CD40 and CD40L, CD28, cytotoxic T-lymphocyte-associated antigen 4 (CTLA-4), B and T lymphocyte attenuator (BTLA), and members of the T cell immunoglobulin and mucin domain (Tim) family. These molecules provide essential co-stimulatory signals for T cell activation and immune homeostasis. Recent studies *in vitro* particularly highlight that excessive interaction between PD-1 and PD-L1 can induce lymphocyte apoptosis, leading to immunosuppression. This immune dysfunction may contribute to tubular epithelial cell damage and the development of septic AKI (45). Consequently, therapeutic strategies aimed at restoring lymphocyte functions such as anti-PD-L1 therapy or lactate receptor blockade, may offer promising avenues for the treatment of sepsis-associated AKI. Drawing on this evidence, it is reasonable to propose that reestablishing the function of immune cells, especially T and B lymphocytes, may play a crucial role in mitigating sepsis-related organ damage and enhancing patient prognosis. The multivariate logistic regression analysis further revealed two distinct cellular patterns associated with AKI risk in sepsis patients: an AKI-promoting pattern and an AKI-protective pattern. Patients with elevated proportions of CD8^+^ T cells expressing inhibitory/exhaustion markers (PD1^+^, TIGIT^+^, TIM3^+^, CCR7^+^CD45RA^+^) and E-MDSCs were more likely to develop AKI, suggesting that immune exhaustion and expansion of immunosuppressive myeloid subsets contribute to renal vulnerability. Conversely, patients with higher levels of checkpoint-positive CD4^+^ T cells (LAG3^+^, CTLA4^+^, TIM3^+^) and specific MDSC subsets (PMN-MDSCs, M-MDSCs) exhibited a lower risk of AKI, indicating that these regulatory mechanisms may buffer excessive immune activation and provide renal protection. While renal impairment and disease severity are well-recognized contributors to AKI, our findings underscore the critical role of immune dysregulation. Taken together, these results suggest that AKI development is tightly linked to an imbalance between pro-injury immune exhaustion and compensatory immunoregulation. This highlights the potential value of targeting immune modulation as a therapeutic strategy to prevent or attenuate AKI in sepsis.

In this study, we identified 13 key predictive factors for AKI risk in sepsis patients using the least absolute shrinkage and selection operator (LASSO) and multivariate logistic regression. The resulting model demonstrated excellent predictive performance, with areas under the curve (AUCs) of 0.989 in the training set and 0.895 in the validation set. Its robustness and accuracy were further validated through five-fold cross-validation, calibration plots, and decision curve analysis, highlighting its potential clinical utility. Compared to previous research, our study presents several distinct advantages. A prior study investigated the association between T-lymphocyte subsets and sepsis-induced AKI, focusing on a limited set of immune parameters (CD3^+^, CD3^+^CD4⁻, CD3^+^CD8^+^, CD4^+^/CD8^+^ ratio, and CD3^+^ percentage) (23). In contrast, our study evaluated 68 types of lymphocyte subsets, including T, B, and natural killer (NK) cells. Furthermore, while the previous study primarily explored the relationship between selected T-lymphocyte subsets and in-hospital mortality, it also reported that CD3^+^ and CD3^+^CD8^+^ T-lymphocyte counts had good predictive value for AKI, with AUCs of 0.849 and 0.856, respectively. In the present study, we developed a comprehensive predictive model for AKI based on lymphocyte subsets measured on both day 1 and day 3 after sepsis onset. Unlike the previous study, we conducted both internal and external validation of our model, further ensuring its reliability. Additionally, our sample size was more than twice that of the earlier study, enhancing the generalizability of our findings. Several other studies have also developed predictive models for sepsis-associated AKI using various approaches. Lin et al. constructed a logistic regression model based on clinical parameters, achieving an AUC of 0.835 in a retrospective analysis (46). Zhang et al. employed a support vector machine model incorporating 43 genes selected via a genetic algorithm, yielding an AUC of 0.948 (47). However, this model lacked external validation. Yue et al. applied the XGBoost algorithm using data from the MIMIC-III database and reported an AUC of 0.821, but similarly did not include a validation cohort (48). Other reported models include logistic regression (49), lightGBM model (50), and pooled analyses without adjustment for confounders (51), with AUCs ranging from 0.712 to 0.873. Compared to these models, our lymphocyte subset-based prediction model demonstrated superior performance and validation, making it a promising tool for the early identification of AKI in sepsis patients.

The present study still has several limitations. First, this is a single-canter, observational study with limited sample size. While we performed rigorous cross-validation and internal validation, external validation with a larger, more diverse cohort is essential to confirm the model’s widespread applicability. Second, although we identified several lymphocyte subsets significantly associated with AKI, their precise role remains to be fully elucidated. It’s unclear whether these cells directly contribute to kidney injury or merely reflect the systemic immune status. Future functional research, incorporating *in vitro* and *in vivo* assays, alongside animal experiments, will be crucial to clarify the causal role of these cell subsets in sepsis-induced AKI. Finally, our analysis did not include certain potentially relevant variables, such as genetic predisposition, detailed hemodynamic parameters, or specific urine biomarkers. Incorporating these factors in future studies could further refine risk prediction and reveal complementary pathophysiological mechanisms.

In conclusion, this study has revealed the unique characteristics of lymphocyte subsets in patients with sepsis-related AKI and successfully constructed a predictive model integrating lymphocyte subsets and clinical indicators, which has good performance and clinical practical value. Future studies should further expand the sample size, strengthen external validation and explore potential molecular mechanisms, with the expectation of promoting the early identification and precise intervention of AKI.

## Data Availability

The original contributions presented in the study are included in the article/**Supplementary Material**, further inquiries can be directed to the corresponding author/s.
