# Supplementary material for: Establishment and validation of the prediction model based on lymphocyte subsets for acute kidney injury in sepsis patients
Source: Front Immunol. 2025 Sep 25;16:1674673. doi: 10.3389/fimmu.2025.1674673 (PMC12507742; doi:10.3389/fimmu.2025.1674673)
Supplement: Supplementary file 1 [file Table1.docx]

**Table S1** Comparisons of the 1^st^ day lymphocyte subsets between non-AKI and AKI groups

| Variables | Non-AKI | AKI | *P* |
| --- | --- | --- | --- |
| nCD64 index | 10.11 (2.00, 31.19) | 6.68 (1.81, 12.32) | **0.022** |
| CD3+T% | 58.56 (48.09, 69.14) | 61.92 (53.30, 72.02) | **0.004** |
| CD4+T% | 31.58 (22.68, 39.10) | 30.42 (22.27, 42.40) | 0.149 |
| CD8+T% | 22.10 (16.24, 29.67) | 23.50 (15.63, 31.97) | 0.292 |
| CD4+CD8+T% | 1.34 (0.71, 2.76) | 1.50 (1.09, 2.09) | 0.502 |
| CD4-CD8-T% | 4.73 (2.11, 8.00) | 3.78 (1.95, 5.82) | 0.097 |
| CD16+CD56+NK% | 14.70 (8.04, 31.68) | 14.99 (8.50, 20.00) | **0.016** |
| CD19+B% | 17.78 (9.02, 27.36) | 16.18 (11.04, 24.46) | 0.426 |
| NKT% | 5.54 (3.20, 10.34) | 4.68 (2.38, 8.16) | **0.021** |
| CD3+T count | 334.00 (167.00, 483.00) | 337.00 (169.00, 777.00) | 0.167 |
| CD4+T count | 152.00 (83.00, 278.00) | 184.00 (84.00, 370.50) | 0.170 |
| CD8+T count | 134.00 (59.00, 225.00) | 109.50 (59.00, 322.00) | 0.637 |
| CD4/CD8 | 1.36 (0.84, 2.30) | 1.44 (0.79, 2.36) | 0.500 |
| CD4+CD8+T count | 4.00 (1.00, 13.00) | 5.00 (2.00, 10.25) | 0.199 |
| CD4-CD8-T count | 11.00 (7.00, 31.00) | 12.00 (5.00, 30.50) | 0.735 |
| CD16+CD56+NK count | 78.00 (43.00, 170.04) | 79.00 (45.50, 120.00) | 0.077 |
| CD19+B count | 110.00 (37.00, 182.00) | 76.00 (42.00, 219.75) | 0.828 |
| Lymphocyte count | 577.00 (383.00, 908.00) | 578.00 (328.00, 1062.75) | 0.802 |
| NKT count | 32.00 (11.00, 73.00) | 23.00 (10.00, 54.75) | 0.108 |
| CD4+CD28+T% | 76.90 (59.20, 89.80) | 85.98 (71.68, 95.95) | **0.002** |
| CD4+CD38+T% | 37.60 (15.80, 55.80) | 55.92 (39.33, 69.36) | **<0.001** |
| CD4+CD69+T% | 73.50 (48.50, 86.50) | 64.30 (46.50, 80.00) | **0.010** |
| CD8+CD28+T% | 38.20 (24.73, 56.70) | 32.30 (21.43, 50.46) | 0.103 |
| CD8+CD38+T% | 35.32 (12.99, 55.70) | 49.50 (35.10, 68.51) | **<0.001** |
| CD8+CD69+T% | 37.10 (23.34, 50.57) | 36.71 (26.60, 52.62) | 0.454 |
| CD155+T% | 43.69 (31.93, 59.79) | 55.95 (34.50, 66.03) | **<0.001** |
| CD4+BTLA+T% | 36.80 (22.20, 65.93) | 34.70 (25.45, 41.80) | 0.438 |
| CD4+CTLA4+T% | 16.25 (10.55, 25.50) | 15.51 (8.98, 26.01) | 0.071 |
| CD4+HLADR+T% | 64.50 (34.98, 96.50) | 78.50 (40.90, 94.30) | 0.169 |
| CD4+LAG3+T% | 43.10 (32.93, 53.60) | 37.30 (23.62, 49.80) | **<0.001** |
| CD4+PD1+T% | 41.71 (24.70, 57.40) | 43.74 (27.10, 53.30) | 0.445 |
| CD4+TIGIT+T% | 51.91 (32.78, 67.08) | 61.04 (26.48, 75.83) | 0.139 |
| CD4+TIM3+T% | 30.98 (18.60, 41.18) | 35.50 (24.39, 43.14) | **0.008** |
| CD4+TcM+T% | 82.40 (69.70, 88.60) | 83.06 (72.01, 89.20) | 0.393 |
| CD4+TeM+T% | 36.77 (12.85, 68.40) | 57.98 (43.12, 75.10) | **<0.001** |
| CD4+TeMRA+T% | 19.82 (1.75, 57.63) | 29.05 (12.31, 63.95) | **0.003** |
| CD4+TN+T% | 99.80 (97.00, 100.00) | 98.16 (81.60, 99.43) | **<0.001** |
| CD8+BTLA+T% | 40.90 (16.60, 61.00) | 38.69 (25.72, 53.12) | 0.762 |
| CD8+CTLA4+T% | 21.64 (13.08, 28.43) | 19.77 (10.80, 31.93) | 0.965 |
| CD8+HLADR+T% | 57.78 (25.30, 84.30) | 68.40 (48.32, 84.20) | **0.005** |
| CD8+LAG3+T% | 35.67 (15.50, 44.70) | 32.68 (15.00, 47.08) | 0.325 |
| CD8+PD1+T% | 28.30 (15.30, 37.50) | 25.80 (17.03, 36.20) | 0.552 |
| CD8+TIGIT+T% | 42.17 (19.20, 61.77) | 61.29 (26.18, 82.28) | **<0.001** |
| CD8+TIM3+T% | 36.50 (19.48, 56.39) | 38.52 (29.04, 46.90) | 0.572 |
| CD8+TcM+T% | 45.37 (24.60, 62.50) | 50.40 (28.07, 71.18) | 0.081 |
| CD8+TeM+T% | 57.60 (42.00, 72.89) | 59.50 (47.22, 80.20) | **0.037** |
| CD8+TeMRA+T% | 81.50 (51.20, 88.80) | 64.23 (47.40, 85.00) | **0.017** |
| CD8+TN+T% | 30.28 (9.75, 68.72) | 26.80 (9.71, 57.90) | 0.083 |
| MDSC | 1.32 (0.28, 7.30) | 2.14 (0.48, 11.82) | **0.046** |
| PMN_MDSC | 0.95 (0.03, 6.00) | 0.29 (0.00, 3.62) | **0.003** |
| M_MDSC | 0.00 (0.00, 0.16) | 0.10 (0.10, 0.72) | **<0.001** |
| e_MDSC | 95.27 (91.08, 98.01) | 97.24 (90.73, 99.02) | **0.004** |
| Th1 | 18.70 (13.55, 25.70) | 19.40 (10.88, 29.70) | 0.335 |
| Th2 | 52.40 (39.02, 67.60) | 56.67 (47.55, 69.28) | 0.120 |
| Th17 | 10.80 (7.45, 17.50) | 10.04 (4.26, 14.62) | **<0.001** |
| Treg | 7.73 (6.35, 11.40) | 8.17 (5.68, 10.75) | 0.757 |
| CD4+CD45RA+T% | 16.25 (6.59, 26.80) | 17.02 (7.91, 28.85) | 0.143 |
| CD4+CD45RO+T% | 59.86 (39.80, 75.08) | 64.60 (54.50, 74.20) | **0.030** |
| CD8+CD45RA+T% | 28.99 (21.38, 41.70) | 39.10 (16.79, 50.50) | **0.006** |
| CD8+CD45RA+T% | 38.07 (18.60, 56.25) | 37.50 (19.40, 50.00) | 0.258 |
| CD4+CCR7+CD45+T% | 21.80 (9.05, 35.40) | 15.50 (10.65, 28.68) | **0.039** |
| CD4+CCR7+CD45-T% | 57.60 (42.28, 70.56) | 51.15 (41.03, 66.64) | **0.016** |
| CD4+CCR7-CD45+T% | 1.59 (0.38, 6.62) | 1.51 (0.49, 3.30) | **0.047** |
| CD4+CCR7-CD45-T% | 8.98 (2.20, 25.50) | 14.25 (5.65, 32.30) | **0.001** |
| CD8+CCR7+CD45+T% | 28.90 (12.79, 42.80) | 28.40 (17.00, 46.20) | 0.719 |
| CD8+CCR7+CD45-T% | 37.30 (25.18, 52.30) | 44.65 (20.85, 65.84) | **0.010** |
| CD8+CCR7-CD45+T% | 8.95 (3.25, 30.00) | 11.25 (4.40, 22.36) | 0.698 |
| CD8+CCR7-CD45-T% | 3.46 (1.19, 15.57) | 5.71 (2.95, 15.30) | **<0.001** |
