# Supplementary material for: Establishment and validation of the prediction model based on lymphocyte subsets for acute kidney injury in sepsis patients
Source: Front Immunol. 2025 Sep 25;16:1674673. doi: 10.3389/fimmu.2025.1674673 (PMC12507742; doi:10.3389/fimmu.2025.1674673)
Supplement: Supplementary file 2 [file Table2.docx]

**Table S2** Comparisons of the 3^rd^ lymphocyte subsets between non-AKI and AKI groups

| **Variables** | **Non-AKI** | **AKI** | ***P*** |
| --- | --- | --- | --- |
| nCD64 index | 4.01 (1.26, 21.44) | 5.92 (2.05, 10.72) | 0.885 |
| CD3+T% | 63.22 (53.81, 73.92) | 62.70 (57.23, 75.25) | 0.126 |
| CD4+T% | 36.02 (28.32, 45.98) | 36.17 (26.44, 48.16) | 0.958 |
| CD8+T% | 22.54 (18.18, 29.95) | 23.99 (16.80, 34.35) | 0.483 |
| CD4+CD8+T% | 1.46 (0.87, 3.14) | 1.35 (0.82, 2.08) | **0.031** |
| CD4-CD8-T% | 3.96 (1.93, 6.61) | 3.86 (1.81, 6.34) | 0.791 |
| CD16+CD56+NK% | 10.28 (5.72, 21.48) | 8.10 (4.98, 16.82) | **<0.001** |
| CD19+B% | 18.48 (11.15, 25.40) | 18.43 (10.90, 31.91) | 0.991 |
| NKT% | 3.84 (2.35, 7.36) | 3.82 (2.53, 8.36) | 0.474 |
| CD3+T count | 637.00 (280.00, 1226.40) | 456.00 (252.00, 957.59) | **0.002** |
| CD4+T count | 331.00 (147.00, 663.00) | 243.00 (146.00, 498.00) | **0.024** |
| CD8+T count | 194.00 (64.00, 353.00) | 164.00 (69.00, 312.00) | 0.691 |
| CD4/CD8 | 1.42 (0.65, 2.10) | 1.83 (0.81, 2.49) | **0.020** |
| CD4+CD8+T count | 7.00 (3.00, 16.00) | 7.50 (2.00, 15.00) | 0.103 |
| CD4-CD8-T count | 16.00 (7.00, 43.00) | 15.00 (6.85, 30.00) | 0.526 |
| CD16+CD56+NK count | 109.00 (52.96, 161.00) | 63.00 (27.00, 117.00) | **<0.001** |
| CD19+B count | 161.00 (61.00, 282.00) | 120.50 (59.50, 204.80) | **0.012** |
| Lymphocyte count | 867.00 (404.00, 1850.00) | 701.00 (432.00, 1463.00) | 0.115 |
| NKT count | 41.00 (11.00, 69.00) | 35.00 (12.00, 73.00) | 0.918 |
| CD4+CD28+T% | 91.90 (48.45, 99.34) | 85.80 (71.87, 96.25) | 0.314 |
| CD4+CD38+T% | 39.30 (12.13, 69.36) | 49.35 (29.80, 68.12) | **0.002** |
| CD4+CD69+T% | 67.60 (37.78, 89.35) | 62.60 (46.40, 80.30) | 0.147 |
| CD8+CD28+T% | 43.60 (29.80, 62.54) | 40.50 (30.80, 67.60) | 0.328 |
| CD8+CD38+T% | 34.40 (5.74, 71.20) | 44.95 (27.27, 64.50) | **0.016** |
| CD8+CD69+T% | 38.01 (23.49, 53.05) | 38.99 (27.40, 54.40) | 0.453 |
| CD155+T% | 45.38 (38.67, 52.46) | 44.90 (35.01, 61.67) | 0.963 |
| CD4+BTLA+T% | 27.00 (13.80, 66.50) | 29.87 (23.07, 49.11) | 0.246 |
| CD4+CTLA4+T% | 30.89 (10.50, 50.24) | 12.19 (3.49, 27.76) | **<0.001** |
| CD4+HLADR+T% | 72.70 (22.57, 97.41) | 54.40 (27.30, 91.35) | **0.047** |
| CD4+LAG3+T% | 45.05 (28.80, 61.50) | 33.70 (26.75, 58.80) | **0.005** |
| CD4+PD1+T% | 37.40 (25.40, 68.31) | 48.55 (33.72, 61.07) | 0.090 |
| CD4+TIGIT+T% | 50.60 (27.94, 69.08) | 57.00 (28.56, 79.12) | 0.050 |
| CD4+TIM3+T% | 39.02 (17.77, 54.33) | 27.45 (13.17, 42.39) | **<0.001** |
| CD4+TcM+T% | 84.38 (77.35, 89.76) | 83.86 (72.27, 93.74) | 0.304 |
| CD4+TeM+T% | 52.55 (21.60, 82.20) | 60.65 (29.28, 82.10) | 0.085 |
| CD4+TeMRA+T% | 9.23 (0.03, 51.98) | 23.03 (5.76, 56.75) | **<0.001** |
| CD4+TN+T% | 98.45 (96.14, 99.70) | 98.93 (96.23, 100.00) | 0.054 |
| CD8+BTLA+T% | 36.36 (18.70, 59.90) | 36.05 (23.58, 48.20) | 0.979 |
| CD8+CTLA4+T% | 22.83 (10.39, 40.49) | 18.90 (7.18, 25.63) | **<0.001** |
| CD8+HLADR+T% | 56.65 (23.86, 87.50) | 66.20 (41.35, 85.03) | **0.045** |
| CD8+LAG3+T% | 18.45 (10.90, 39.78) | 29.99 (13.77, 40.48) | **0.002** |
| CD8+PD1+T% | 16.90 (12.30, 25.89) | 25.85 (17.14, 37.10) | **<0.001** |
| CD8+TIGIT+T% | 35.39 (15.65, 70.45) | 71.43 (18.72, 87.46) | **<0.001** |
| CD8+TIM3+T% | 42.84 (29.76, 59.88) | 32.49 (15.16, 45.62) | **<0.001** |
| CD8+TcM+T% | 62.87 (30.42, 79.60) | 59.38 (36.03, 76.31) | 0.233 |
| CD8+TeM+T% | 53.14 (37.90, 68.91) | 55.80 (39.82, 72.50) | 0.556 |
| CD8+TeMRA+T% | 71.53 (52.57, 89.40) | 69.22 (43.52, 87.80) | **<0.001** |
| CD8+TN+T% | 40.94 (19.07, 69.00) | 50.50 (16.73, 77.35) | **0.024** |
| MDSC | 3.83 (0.57, 14.61) | 1.53 (0.50, 7.39) | **<0.001** |
| PMN_MDSC | 0.00 (0.00, 0.44) | 0.18 (0.01, 1.00) | **<0.001** |
| M_MDSC | 0.00 (0.00, 0.71) | 0.29 (0.00, 1.78) | **<0.001** |
| e_MDSC | 99.92 (54.79, 100.00) | 98.29 (93.22, 99.60) | **<0.001** |
| Th1 | 26.70 (16.02, 37.98) | 20.21 (13.20, 36.78) | **0.037** |
| Th2 | 52.45 (40.72, 65.93) | 54.77 (38.34, 64.20) | 0.837 |
| Th17 | 8.86 (6.06, 12.30) | 8.03 (3.64, 12.58) | 0.185 |
| Treg | 9.12 (7.35, 11.45) | 9.29 (7.41, 11.38) | 0.141 |
| CD4+CD45RA+T% | 8.25 (1.66, 24.26) | 16.30 (10.26, 33.53) | **<0.001** |
| CD4+CD45RO+T% | 61.70 (39.80, 83.60) | 65.80 (42.95, 78.30) | 0.844 |
| CD8+CD45RA+T% | 21.53 (6.86, 43.73) | 32.75 (19.75, 56.10) | **<0.001** |
| CD8+CD45RA+T% | 35.70 (25.70, 50.35) | 35.20 (20.71, 49.25) | 0.454 |
| CD4+CCR7+CD45+T% | 18.88 (6.68, 39.78) | 22.40 (12.60, 38.41) | 0.308 |
| CD4+CCR7+CD45-T% | 58.70 (44.97, 72.23) | 58.95 (47.32, 69.50) | 0.700 |
| CD4+CCR7-CD45+T% | 0.66 (0.01, 2.79) | 1.81 (0.65, 3.03) | **<0.001** |
| CD4+CCR7-CD45-T% | 9.29 (2.51, 26.21) | 9.40 (3.56, 17.90) | 0.946 |
| CD8+CCR7+CD45+T% | 14.34 (7.96, 38.00) | 33.00 (14.70, 44.50) | **<0.001** |
| CD8+CCR7+CD45-T% | 36.95 (23.14, 51.30) | 39.45 (21.60, 58.73) | 0.235 |
| CD8+CCR7-CD45+T% | 9.89 (2.60, 26.70) | 10.90 (4.04, 25.84) | 0.556 |
| CD8+CCR7-CD45-T% | 19.37 (3.79, 40.30) | 4.91 (1.45, 23.10) | **<0.001** |
