# Supplementary material for: Establishment and validation of the prediction model based on lymphocyte subsets for acute kidney injury in sepsis patients
Source: Front Immunol. 2025 Sep 25;16:1674673. doi: 10.3389/fimmu.2025.1674673 (PMC12507742; doi:10.3389/fimmu.2025.1674673)
Supplement: Supplementary file 3 [file Table3.docx]

**Table S3** Univariate logistic regression for clinical characteristics and lymphocyte subsets in sepsis patients

| **Variables** | **β** | **S.E** | **Z** | ***P*** | **OR (95%CI)** |
| --- | --- | --- | --- | --- | --- |
| Gender (Male vs female) | -0.350 | 0.329 | -1.066 | 0.287 | 0.705 (0.370-1.342) |
| Age | 0.000 | 0.010 | -0.037 | 0.970 | 1.000 (0.980-1.020) |
| BMI | 0.025 | 0.039 | 0.647 | 0.518 | 1.026 (0.950-1.107) |
| SOFA | 0.161 | 0.048 | 3.372 | **<0.001** | 1.174 (1.070-1.289) |
| APACHEII | 0.107 | 0.024 | 4.430 | **<0.001** | 1.113 (1.062-1.168) |
| GCS | -0.103 | 0.045 | -2.294 | **0.022** | 0.902 (0.826-0.985) |
| COPD (Yes vs No) | 17.500 | 1251.054 | 0.014 | 0.989 | 39817580.764 (0.000-Inf) |
| Hypertension (Yes vs No) | -0.407 | 0.328 | -1.240 | 0.215 | 0.666 (0.350-1.266) |
| Diabetes (Yes vs No) | -0.198 | 0.352 | -0.563 | 0.574 | 0.820 (0.411-1.635) |
| CHD (Yes vs No) | 0.131 | 0.341 | 0.382 | 0.702 | 1.139 (0.584-2.224) |
| CRRT (Yes vs No) | 0.937 | 0.362 | 2.587 | **0.010** | 2.553 (1.255-5.195) |
| Vasoactive drugs (Yes vs No) | 0.976 | 0.347 | 2.813 | **0.005** | 2.654 (1.344-5.238) |
| Septic shock (Yes vs No) | 1.237 | 0.358 | 3.455 | **<0.001** | 3.446 (1.708-6.951) |
| Primary Infection (Yes vs No) | 1.183 | 0.558 | 2.119 | **0.034** | 3.266 (1.093-9.755) |
| Whtie blood cell, 109/L | 0.033 | 0.020 | 1.646 | 0.100 | 1.034 (0.994-1.076) |
| Hemoglobin, g/L | -0.006 | 0.005 | -1.134 | 0.257 | 0.994 (0.984-1.004) |
| Platelet, 109/L | -0.001 | 0.002 | -0.436 | 0.663 | 0.999 (0.996-1.002) |
| Neutrophil, 109/L | 0.025 | 0.013 | 1.889 | 0.059 | 1.025 (0.999- 1.051) |
| Lymphocyte, 109/L | 0.221 | 0.139 | 1.593 | 0.111 | 1.248 (0.950-1.638) |
| Monocyte, 109/L | 0.865 | 0.340 | 2.546 | **0.011** | 2.376 (1.220- 4.625) |
| C-reactive protein, mg/L | 0.000 | 0.002 | 0.252 | 0.801 | 1.000 (0.997-1.004) |
| Procalcitonin, ng/L | 0.007 | 0.005 | 1.465 | 0.143 | 1.007 (0.998- 1.017) |
| Total bilirubin, μmol/L | -0.002 | 0.004 | -0.611 | 0.541 | 0.998 (0.991-1.005) |
| Albumin, g/L | -0.024 | 0.026 | -0.943 | 0.346 | 0.976 (0.928-1.027) |
| Globulin, g/L | 0.012 | 0.032 | 0.373 | 0.709 | 1.012 (0.951-1.077) |
| Blood urea nitrogen, mmol/L | 0.030 | 0.016 | 1.893 | 0.058 | 1.031 (0.999-1.064) |
| Creatinine, μmol/L | 0.005 | 0.002 | 3.374 | **<0.001** | 1.005 (1.002-1.008) |
| **First day** |  |  |  |  |  |
| nCD64 index | -0.018 | 0.009 | -2.065 | **0.039** | 0.982 (0.965-0.999) |
| CD3+T% | 0.022 | 0.011 | 1.970 | **0.049** | 1.022 (1.001-1.044) |
| CD4+T% | -0.001 | 0.001 | -0.582 | 0.560 | 0.999 (0.997-1.002) |
| CD8+T% | -0.052 | 0.082 | -0.636 | 0.525 | 0.949 (0.809-1.114) |
| CD4+CD8+T% | 0.012 | 0.013 | 0.913 | 0.361 | 1.012 (0.986- 1.039) |
| CD4-CD8-T% | -0.005 | 0.025 | -0.198 | 0.843 | 0.995 (0.948-1.045) |
| CD16+CD56+NK% | -0.026 | 0.012 | -2.086 | **0.037** | 0.974 (0.951-0.998) |
| CD19+B% | -0.010 | 0.013 | -0.755 | 0.450 | 0.990 (0.965-1.016) |
| NKT% | -0.008 | 0.020 | -0.400 | 0.689 | 0.992 (0.954-1.032) |
| CD3+T count | 0.000 | 0.000 | 0.245 | 0.807 | 1.000 (1.000-1.001) |
| CD4+T count | 0.000 | 0.000 | -0.086 | 0.931 | 1.000 (0.999-1.001) |
| CD8+T count | 0.000 | 0.001 | 0.704 | 0.482 | 1.000 (0.999-1.002) |
| CD4/CD8 | 0.039 | 0.102 | 0.385 | 0.700 | 1.040 (0.851-1.271) |
| CD4+CD8+T count | 0.002 | 0.009 | 0.191 | 0.849 | 1.002 (0.984-1.020) |
| CD4-CD8-T count | 0.000 | 0.002 | -0.199 | 0.843 | 1.000 (0.995-1.004) |
| CD16+CD56+T count | -0.001 | 0.001 | -0.958 | 0.338 | 0.999 (0.997-1.001) |
| CD19+B count | 0.000 | 0.001 | 0.043 | 0.965 | 1.000 (0.998- 1.002) |
| lymphocyte count | 0.000 | 0.000 | 0.020 | 0.984 | 1.000 (1.000-1.000) |
| NKT count | 0.000 | 0.001 | 0.048 | 0.962 | 1.000 (0.997-1.003) |
| CD4+CD28+T% | 0.008 | 0.007 | 1.135 | 0.256 | 1.008 (0.995-1.021) |
| CD4+CD38+T% | 0.023 | 0.006 | 3.505 | **<0.001** | 1.023 (1.010-1.036) |
| CD4+CD69+T% | -0.009 | 0.007 | -1.257 | 0.209 | 0.991 (0.976-1.005) |
| CD8+CD28+T% | -0.005 | 0.007 | -0.613 | 0.540 | 0.995 (0.981-1.010) |
| CD8+CD38+T% | 0.021 | 0.006 | 3.227 | **0.001** | 1.021 (1.008-1.034) |
| CD8+CD69+T% | 0.004 | 0.008 | 0.479 | 0.632 | 1.004 (0.988-1.019) |
| CD155+T% | 0.018 | 0.008 | 2.167 | **0.030** | 1.018 (1.002-1.035) |
| CD4+BTLA+T% | -0.007 | 0.007 | -0.953 | 0.341 | 0.993 (0.979-1.007) |
| CD4+CTLA4+T% | -0.011 | 0.013 | -0.798 | 0.425 | 0.989 (0.963-1.016) |
| CD4+HLADR+T% | 0.004 | 0.005 | 0.775 | 0.439 | 1.004 (0.994-1.014) |
| CD4+LAG3+T% | -0.018 | 0.009 | -2.108 | **0.035** | 0.982 (0.966-0.999) |
| CD4+PD1+T% | 0.003 | 0.008 | 0.420 | 0.674 | 1.003 (0.988-1.019) |
| CD4+TIGIT+T% | 0.002 | 0.005 | 0.374 | 0.709 | 1.002 (0.991-1.013) |
| CD4+TIM3+T% | 0.009 | 0.010 | 0.934 | 0.350 | 1.010 (0.990-1.030) |
| CD4+TcM+T% | 0.004 | 0.009 | 0.513 | 0.608 | 1.004 (0.987-1.022) |
| CD4+TeM+T% | 0.020 | 0.006 | 3.389 | **<0.001** | 1.020 (1.008-1.032) |
| CD4+TeMRA+T% | 0.007 | 0.005 | 1.414 | 0.157 | 1.007 (0.997-1.017) |
| CD4+TN+T% | -0.020 | 0.011 | -1.904 | 0.057 | 0.980 (0.960-1.001) |
| CD8+BTLA+T% | -0.002 | 0.008 | -0.265 | 0.791 | 0.998 (0.983-1.013) |
| CD8+CTLA4+T% | 0.008 | 0.013 | 0.612 | 0.541 | 1.008 (0.983-1.033) |
| CD8+HLADR+T% | 0.009 | 0.006 | 1.638 | 0.101 | 1.009 (0.998-1.020) |
| CD8+LAG3+T% | -0.009 | 0.008 | -1.085 | 0.278 | 0.991 (0.976-1.007) |
| CD8+PD1+T% | -0.003 | 0.010 | -0.316 | 0.752 | 0.997 (0.976-1.017) |
| CD8+TIGIT+T% | 0.012 | 0.005 | 2.264 | **0.024** | 1.012 (1.002-1.022) |
| CD8+TIM3+T% | 0.000 | 0.009 | -0.012 | 0.990 | 1.000 (0.983-1.018) |
| CD8+TcM+T% | 0.007 | 0.007 | 1.055 | 0.291 | 1.007 (0.994-1.020) |
| CD8+TeM+T% | 0.013 | 0.008 | 1.592 | 0.111 | 1.013 (0.997-1.030) |
| CD8+TeMRA+T% | -0.009 | 0.007 | -1.219 | 0.223 | 0.992 (0.978- 1.005) |
| CD8+TN+T% | -0.005 | 0.005 | -1.016 | 0.310 | 0.995 (0.985-1.005) |
| MDSC | 0.004 | 0.014 | 0.294 | 0.769 | 1.004 (0.978-1.031) |
| PMN_MDSC | 0.030 | 0.025 | 1.174 | 0.240 | 1.030 (0.980-1.082) |
| M_MDSC | 0.042 | 0.032 | 1.311 | 0.190 | 1.043 (0.980- 1.110) |
| e_MDSC | 0.000 | 0.022 | 0.013 | 0.990 | 1.000 (0.958-1.045) |
| Th1 | 0.017 | 0.012 | 1.417 | 0.156 | 1.017 (0.993-1.042) |
| Th2 | 0.009 | 0.009 | 0.998 | 0.318 | 1.009 (0.991-1.028) |
| Th17 | -0.052 | 0.022 | -2.414 | **0.016** | 0.949 (0.910- 0.990) |
| Treg | -0.021 | 0.045 | -0.472 | 0.637 | 0.979 (0.896- 1.070) |
| CD4+CD45RA+T% | 0.011 | 0.011 | 1.075 | 0.282 | 1.011 (0.991-1.033) |
| CD4+CD45RO+T% | 0.006 | 0.007 | 0.817 | 0.414 | 1.006 (0.992-1.019) |
| CD8+CD45RA+T% | 0.013 | 0.008 | 1.731 | 0.083 | 1.013 (0.998-1.028) |
| CD8+CD45RA+T% | 0.006 | 0.008 | 0.842 | 0.400 | 1.006 (0.991-1.022) |
| CD4+CCR7+CD45+T% | -0.012 | 0.009 | -1.292 | 0.196 | 0.988 (0.970- 1.006) |
| CD4+CCR7+CD45-T% | -0.011 | 0.009 | -1.242 | 0.214 | 0.989 (0.972-1.006) |
| CD4+CCR7-CD45+T% | -0.091 | 0.035 | -2.597 | **0.009** | 0.913 (0.852-0.978) |
| CD4+CCR7-CD45-T% | 0.013 | 0.008 | 1.542 | 0.123 | 1.013 (0.996-1.030) |
| CD8+CCR7+CD45+T% | 0.001 | 0.008 | 0.105 | 0.916 | 1.001 (0.985- 1.017) |
| CD8+CCR7+CD45-T% | 0.012 | 0.007 | 1.752 | 0.080 | 1.012 (0.999-1.026) |
| CD8+CCR7-CD45+T% | -0.010 | 0.008 | -1.159 | 0.247 | 0.990 (0.975- 1.007) |
| CD8+CCR7-CD45-T% | 0.016 | 0.011 | 1.415 | 0.157 | 1.016 (0.994-1.038) |
| **Third day** |  |  |  |  |  |
| nCD64 index | -0.023 | 0.012 | -1.905 | 0.057 | 0.977 (0.953-1.001) |
| CD3+T% | 0.012 | 0.012 | 0.949 | 0.343 | 1.012 (0.988-1.037) |
| CD4+T% | 0.001 | 0.013 | 0.109 | 0.913 | 1.001 (0.977-1.027) |
| CD8+T% | 0.014 | 0.015 | 0.965 | 0.334 | 1.015 (0.985-1.045) |
| CD4+CD8+T% | -0.210 | 0.100 | -2.090 | 0.037 | 0.811 (0.666- 0.987) |
| CD4-CD8-T% | 0.000 | 0.041 | -0.001 | 1.000 | 1.000 (0.923-1.083) |
| CD16+CD56+NK% | -0.041 | 0.017 | -2.403 | **0.016** | 0.960 (0.929-0.993) |
| CD19+B% | 0.008 | 0.013 | 0.637 | 0.524 | 1.008 (0.983-1.035) |
| NKT% | 0.017 | 0.027 | 0.630 | 0.529 | 1.017 (0.965-1.071) |
| CD3+T count | 0.000 | 0.000 | -1.901 | 0.057 | 1.000 (0.999- 1.000) |
| CD4+T count | -0.001 | 0.000 | -1.852 | 0.064 | 0.999 (0.998-1.000) |
| CD8+T count | 0.000 | 0.001 | -0.146 | 0.884 | 1.000 (0.999-1.001) |
| CD4/CD8 | -0.013 | 0.018 | -0.727 | 0.467 | 0.987 (0.953- 1.023) |
| CD4+CD8+T count | -0.004 | 0.010 | -0.336 | 0.737 | 0.996 (0.976-1.017) |
| CD4-CD8-T count | -0.002 | 0.003 | -0.591 | 0.555 | 0.998 (0.991-1.005) |
| CD16+CD56+T count | -0.004 | 0.002 | -2.336 | **0.019** | 0.996 (0.992- 0.999) |
| CD19+B count | -0.001 | 0.001 | -1.515 | 0.130 | 0.999 (0.997-1.000) |
| lymphocyte count | 0.000 | 0.000 | -0.820 | 0.412 | 1.000 (1.000- 1.000) |
| NKT count | -0.001 | 0.002 | -0.485 | 0.628 | 0.999 (0.994-1.004) |
| CD4+CD28+T% | 0.013 | 0.006 | 2.036 | **0.042** | 1.013 (1.001-1.025) |
| CD4+CD38+T% | 0.010 | 0.006 | 1.799 | 0.072 | 1.010 (0.999-1.021) |
| CD4+CD69+T% | -0.005 | 0.006 | -0.769 | 0.442 | 0.995 (0.983-1.008) |
| CD8+CD28+T% | 0.006 | 0.008 | 0.776 | 0.438 | 1.006 (0.991-1.022) |
| CD8+CD38+T% | 0.006 | 0.005 | 1.046 | 0.295 | 1.006 (0.995-1.016) |
| CD8+CD69+T% | -0.001 | 0.007 | -0.079 | 0.937 | 0.999 (0.985-1.014) |
| CD155+T% | 0.012 | 0.012 | 1.038 | 0.299 | 1.012 (0.989-1.035) |
| CD4+BTLA+T% | -0.003 | 0.006 | -0.465 | 0.642 | 0.997 (0.985-1.009) |
| CD4+CTLA4+T% | -0.038 | 0.009 | -4.291 | **<0.001** | 0.962 (0.946- 0.979) |
| CD4+HLADR+T% | -0.004 | 0.004 | -0.844 | 0.398 | 0.996 (0.988-1.005) |
| CD4+LAG3+T% | -0.013 | 0.007 | -1.730 | 0.084 | 0.987 (0.973-1.002) |
| CD4+PD1+T% | 0.006 | 0.008 | 0.773 | 0.440 | 1.006 (0.991-1.021) |
| CD4+TIGIT+T% | 0.006 | 0.006 | 0.963 | 0.335 | 1.006 (0.994-1.019) |
| CD4+TIM3+T% | -0.029 | 0.009 | -3.119 | **0.002** | 0.971 (0.954-0.989) |
| CD4+TcM+T% | -0.016 | 0.011 | -1.406 | 0.160 | 0.985 (0.963-1.006) |
| CD4+TeM+T% | 0.005 | 0.005 | 1.028 | 0.304 | 1.005 (0.995-1.016) |
| CD4+TeMRA+T% | 0.009 | 0.005 | 1.643 | 0.100 | 1.009 (0.998-1.019) |
| CD4+TN+T% | -0.053 | 0.039 | -1.352 | 0.176 | 0.948 (0.878- 1.024) |
| CD8+BTLA+T% | -0.003 | 0.008 | -0.422 | 0.673 | 0.997 (0.982-1.012) |
| CD8+CTLA4+T% | -0.042 | 0.012 | -3.559 | **<0.001** | 0.959 (0.937- 0.981) |
| CD8+HLADR+T% | 0.009 | 0.005 | 1.669 | 0.095 | 1.009 (0.998-1.020) |
| CD8+LAG3+T% | 0.012 | 0.008 | 1.463 | 0.143 | 1.012 (0.996-1.029) |
| CD8+PD1+T% | 0.046 | 0.014 | 3.338 | **<0.001** | 1.047 (1.019- 1.075) |
| CD8+TIGIT+T% | 0.015 | 0.005 | 2.867 | **0.004** | 1.015 (1.005-1.026) |
| CD8+TIM3+T% | -0.041 | 0.010 | -4.208 | **<0.001** | 0.960 (0.942-0.978) |
| CD8+TcM+T% | -0.004 | 0.006 | -0.675 | 0.499 | 0.996 (0.984- 1.008) |
| CD8+TeM+T% | 0.003 | 0.008 | 0.366 | 0.714 | 1.003 (0.988-1.018) |
| CD8+TeMRA+T% | -0.014 | 0.007 | -1.969 | **0.049** | 0.986 (0.973-0.999) |
| CD8+TN+T% | 0.007 | 0.005 | 1.256 | 0.209 | 1.007 (0.996-1.017) |
| MDSC | -0.047 | 0.023 | -2.064 | **0.039** | 0.955 (0.913- 0.998) |
| PMN_MDSC | -0.078 | 0.034 | -2.282 | **0.023** | 0.925 (0.866- 0.989) |
| M_MDSC | -0.042 | 0.020 | -2.050 | **0.040** | 0.959 (0.922- 0.998) |
| e_MDSC | 0.035 | 0.010 | 3.442 | **<0.001** | 1.036 (1.015-1.056) |
| Th1 | -0.005 | 0.010 | -0.513 | 0.608 | 0.995 (0.975- 1.015) |
| Th2 | 0.000 | 0.010 | 0.040 | 0.968 | 1.000 (0.981- 1.020) |
| Th17 | -0.005 | 0.026 | -0.193 | 0.847 | 0.995 (0.946- 1.047) |
| Treg | 0.047 | 0.046 | 1.027 | 0.304 | 1.048 (0.958-1.147) |
| CD4+CD45RA+T% | 0.017 | 0.009 | 1.946 | 0.052 | 1.018 (1.000-1.036) |
| CD4+CD45RO+T% | 0.001 | 0.006 | 0.201 | 0.840 | 1.001 (0.989-1.014) |
| CD8+CD45RA+T% | 0.029 | 0.008 | 3.528 | **<0.001** | 1.029 (1.013- 1.045) |
| CD8+CD45RA+T% | 0.000 | 0.008 | -0.031 | 0.975 | 1.000 (0.984-1.016) |
| CD4+CCR7+CD45+T% | 0.008 | 0.008 | 1.039 | 0.299 | 1.008 (0.993-1.025) |
| CD4+CCR7+CD45-T% | -0.004 | 0.008 | -0.470 | 0.638 | 0.996 (0.980-1.013) |
| CD4+CCR7-CD45+T% | -0.057 | 0.044 | -1.290 | 0.197 | 0.945 (0.867-1.030) |
| CD4+CCR7-CD45-T% | -0.007 | 0.008 | -0.789 | 0.430 | 0.993 (0.977-1.010) |
| CD8+CCR7+CD45+T% | 0.022 | 0.008 | 2.703 | **0.007** | 1.022 (1.006-1.038) |
| CD8+CCR7+CD45-T% | 0.005 | 0.007 | 0.712 | 0.476 | 1.005 (0.991-1.020) |
| CD8+CCR7-CD45+T% | -0.004 | 0.008 | -0.531 | 0.595 | 0.996 (0.980-1.012) |
| CD8+CCR7-CD45-T% | -0.027 | 0.009 | -2.911 | **0.004** | 0.973 (0.955-0.991) |
