# Supplementary material for: Establishment and validation of the prediction model based on lymphocyte subsets for acute kidney injury in sepsis patients
Source: Front Immunol. 2025 Sep 25;16:1674673. doi: 10.3389/fimmu.2025.1674673 (PMC12507742; doi:10.3389/fimmu.2025.1674673)
Supplement: Supplementary file 4 [file Table4.docx]

**Table S4** Comparison of clinical characteristics among the training, validation and test sets

| **Variables** | **Training set** | **Validation set** | **Test set** | ***P*** |
| --- | --- | --- | --- | --- |
| Age, year | 66.02 ± 15.49 | 61.85 ± 16.68 | 65.00 ± 16.79 | 0.784 |
| Gender, Male (%) | 306 (63.35) | 51 (34.93) | 56 (44.80) | 0.184 |
| BMI, kg/m^2^ | 25.05 ± 4.14 | 24.99 ± 3.66 | 24.71 ± 3.44 | 0.698 |
| SOFA | 8.01 ± 3.72 | 7.86 ± 3.80 | 8.06 ± 3.76 | 0.891 |
| APACHEII | 20.20 ± 7.91 | 20.03 ± 8.16 | 20.80 ± 8.39 | 0.704 |
| GCS | 10.78 ± 3.73 | 10.65 ± 3.98 | 11.08 ± 3.39 | 0.609 |
| COPD, n (%) | 30 (6.21) | 9 (6.16) | 6 (4.80) | 0.833 |
| Hypertension, n (%) | 180 (37.27) | 51 (34.93) | 47 (37.60) | 0.862 |
| Diabetes, n (%) | 135 (27.95) | 42 (28.77) | 38 (30.40) | 0.862 |
| CHD, n (%) | 153 (31.68) | 44 (30.14) | 35 (28.00) | 0.717 |
| CRRT, n (%) | 150 (31.06) | 42 (28.77) | 40 (32.00) | 0.826 |
| Vasoactive drugs, n (%) | 327(67.70) | 94 (64.38) | 90 (72.00) | 0.408 |
| Septic shock, n (%) | 333 (68.94) | 99 (67.81) | 93 (74.40) | 0.431 |
| Primary Infection, n (%) | 432 (89.44) | 133 (91.10) | 115 (92.00) | 0.636 |
| Whtie blood cell, 10^9^/L | 14.07 ± 8.12 | 12.90 ± 7.25 | 13.17 ± 7.50 | 0.207 |
| Hemoglobin, g/L | 110.36 ± 30.66 | 112.67 ± 31.09 | 106.55 ± 31.25 | 0.259 |
| Platelet, 10^9^/L | 167.60 ± 104.54 | 156.78 ± 89.02 | 161.74 ± 96.60 | 0.492 |
| Neutrophil, 10^9^/L | 16.98 ± 24.16 | 17.68 ± 28.50 | 17.65 ± 29.25 | 0.940 |
| Lymphocyte, 10^9^/L | 1.33 ± 2.55 | 1.39 ± 2.43 | 1.00 ± 1.24 | 0.315 |
| Monocyte, 10^9^/L | 0.70 ± 0.74 | 0.64 ± 0.64 | 0.60 ± 0.47 | 0.263 |
| C-reactive protein, mg/L | 137.03 ± 96.00 | 132.17 ± 97.19 | 132.67 ± 97.42 | 0.818 |
| Procalcitonin, ng/L | 24.67 ± 32.65 | 19.66 ± 27.29 | 26.19 ± 33.84 | 0.173 |
| Total bilirubin, μmol/L | 40.47 ± 45.22 | 43.32 ± 49.13 | 44.51 ± 48.89 | 0.617 |
| Albumin, g/L | 30.07 ± 6.18 | 30.35 ± 6.29 | 30.56 ± 6.50 | 0.700 |
| Globulin, g/L | 26.95 ± 4.99 | 26.93 ± 5.35 | 27.61 ± 5.34 | 0.414 |
| Blood urea nitrogen, mmol/L | 16.38 ± 11.15 | 15.71 ± 10.65 | 17.94 ± 11.12 | 0.232 |
| Creatinine, μmol/L | 200.84 ± 221.14 | 176.32 ± 184.04 | 221.78 ± 232.93 | 0.221 |
| Lactate, mmol/L | 4.30 ± 13.81 | 5.64 ± 20.24 | 4.03 ± 3.31 | 0.557 |
