# Supplementary material for: Establishment and validation of the prediction model based on lymphocyte subsets for acute kidney injury in sepsis patients
Source: Front Immunol. 2025 Sep 25;16:1674673. doi: 10.3389/fimmu.2025.1674673 (PMC12507742; doi:10.3389/fimmu.2025.1674673)
Supplement: Supplementary file 5 [file Table5.docx]

**Table S5** Comparisons of the 1^st^ lymphocyte subsets among training, validation and test sets

| **Variables** | **Training set** | **Validation set** | **Test set** | ***P*** |
| --- | --- | --- | --- | --- |
| nCD64 index | 7.69 (1.8120.53) | 9.12 (2.71, 20.23) | 9.23 (4.11,20.53) | 0.472 |
| CD3+T% | 60.67 (50.3471.42) | 60.67 (47.68, 71.42) | 60.22 (51.18,70.67) | 0.969 |
| CD4+T% | 30.42 (22.5241.01) | 27.58 (22.27, 38.94) | 29.88 (21.64,39.08) | 0.221 |
| CD8+T% | 22.32 (15.7431.17) | 23.30 (15.89, 33.18) | 22.32 (15.74,33.17) | 0.742 |
| CD4+CD8+T% | 1.44 (0.882.28) | 1.44 (0.92, 2.56) | 1.45 (1.02,2.28) | 0.842 |
| CD4-CD8-T% | 3.86 (1.967.63) | 4.56 (2.53, 8.48) | 4.73 (2.52,8.19) | 0.100 |
| CD16+CD56+NK% | 14.70 (8.0424.13) | 14.25 (7.33, 27.81) | 14.70 (8.04,25.78) | 0.968 |
| CD19+B% | 17.60 (9.9226.52) | 17.56 (9.92, 26.41) | 17.52 (9.38,26.52) | 0.931 |
| NKT% | 5.10 (2.669.07) | 5.76 (3.09, 10.83) | 5.20(2.38,9.74) | 0.339 |
| CD3+T count | 337.00 (169.00633.00) | 330.50 (184.50, 631.00) | 334.00(169.00,592.00) | 0.956 |
| CD4+T count | 173.00 (84.00332.00) | 149.50 (85.25, 304.50) | 173.00 (84.00,366.00) | 0.699 |
| CD8+T count | 131.00 (59.00231.00) | 141.50 (59.50, 221.50) | 134.00 (59.00,226.00) | 0.943 |
| CD4/CD8 | 1.43 (0.792.34) | 1.26 (0.73, 2.13) | 1.41 (0.74,2.34) | 0.394 |
| CD4+CD8+T count | 5.00 (2.0011.00) | 5.00 (2.00, 11.00) | 6.00 (2.00,13.00) | 0.701 |
| CD4-CD8-T count | 12.00 (6.0031.00) | 15.50 (6.00, 37.00) | 14.00(6.00,31.00) | 0.520 |
| CD16+CD56+T count | 78.00 (44.00145.00) | 81.50 (49.00, 145.00) | 83.00 (48.00,147.00) | 0.913 |
| CD19+B count | 82.00 (40.86201.00) | 77.00 (40.86, 182.00) | 77.000(42.00,208.00) | 0.815 |
| lymphocyte count | 577.00 (352.001043.00) | 537.00 (359.50, 1021.00) | 560.00(359.00,1122.00) | 0.842 |
| NKT count | 27.00 (11.0066.00) | 33.00 (11.00, 67.00) | 26.00 (10.00,66.00) | 0.705 |
| CD4+CD28+T% | 84.50 (65.7393.70) | 84.50 (67.40, 93.70) | 85.91 (69.52,96.70) | 0.541 |
| CD4+CD38+T% | 51.20 (22.7766.00) | 48.12 (18.99, 63.02) | 52.40 (24.10,66.69) | 0.341 |
| CD4+CD69+T% | 67.26 (46.5085.20) | 64.40 (42.80, 88.20) | 67.26 (46.00,84.60) | 0.923 |
| CD8+CD28+T% | 34.02 (23.9053.80) | 37.41 (21.68, 49.87) | 39.50 (26.70,61.23) | 0.084 |
| CD8+CD38+T% | 44.80 (22.8062.75) | 44.80 (14.71, 62.22) | 45.12 (20.79,62.75) | 0.751 |
| CD8+CD69+T% | 36.71 (23.5050.77) | 36.39 (25.88, 51.77) | 36.71 (23.50,52.10) | 0.971 |
| CD155+T% | 46.64 (33.2163.00) | 45.60 (34.02, 62.90) | 46.64 (33.21,63.00) | 0.975 |
| CD4+BTLA+T% | 35.70 (24.6056.78) | 34.55 (24.50, 56.66) | 33.90 (24.50,51.70) | 0.812 |
| CD4+CTLA4+T% | 15.60 (9.3726.01) | 15.46 (8.39, 23.56) | 16.25 (9.48,26.01) | 0.417 |
| CD4+HLADR+T% | 68.00 (36.8094.90) | 69.60 (44.34, 96.60) | 65.80 (34.98,94.30) | 0.538 |
| CD4+LAG3+T% | 39.42 (26.5052.07) | 37.15 (23.70, 50.62) | 39.42 (25.63,52.07) | 0.347 |
| CD4+PD1+T% | 42.60 (25.9055.29) | 37.90 (25.72, 54.82) | 41.71(25.90,55.46) | 0.708 |
| CD4+TIGIT+T% | 57.10 (29.1575.60) | 54.66 (36.89, 74.70) | 62.68 (40.63,79.00) | 0.210 |
| CD4+TIM3+T% | 33.70 (22.0942.85) | 35.24 (19.96, 42.89) | 35.19 (21.84,40.94) | 0.986 |
| CD4+TcM+T% | 82.70 (70.8689.20) | 83.50 (72.40, 88.50) | 83.60 (73.90,90.40) | 0.482 |
| CD4+TeM+T% | 50.30 (23.9574.40) | 52.44 (20.73, 78.57) | 49.10 (20.22,77.47) | 0.928 |
| CD4+TeMRA+T% | 25.40 (7.2162.70) | 26.70 (8.79, 57.63) | 31.20 (5.00,67.70) | 0.940 |
| CD4+TN+T% | 98.70 (94.40100.00) | 98.70 (94.73, 99.98) | 99.200(94.10,100.00) | 0.510 |
| CD8+BTLA+T% | 38.69 (22.0054.90) | 38.69 (18.77, 55.50) | 38.80 (20.42,61.62) | 0.849 |
| CD8+CTLA4+T% | 20.71 (10.9629.56) | 17.30 (10.02, 29.35) | 18.75 (11.34,29.35) | 0.493 |
| CD8+HLADR+T% | 64.83 (34.9884.30) | 53.95 (30.70, 81.23) | 65.30 (40.92,84.80) | 0.121 |
| CD8+LAG3+T% | 32.68 (15.3046.50) | 32.68 (18.90, 44.10) | 33.026 (15.300,46.596) | 0.845 |
| CD8+PD1+T% | 26.20 (16.4037.19) | 24.50 (16.11, 35.63) | 24.500 (16.400,35.800) | 0.540 |
| CD8+TIGIT+T% | 48.65 (20.4877.50) | 49.39 (28.38, 77.27) | 48.372 (26.175,77.177) | 0.978 |
| CD8+TIM3+T% | 38.01 (26.3950.12) | 37.90 (23.89, 50.40) | 38.008 (26.724,47.600) | 0.970 |
| CD8+TcM+T% | 47.20 (26.4068.00) | 45.84 (28.08, 62.78) | 47.770 (26.900,67.800) | 0.881 |
| CD8+TeM+T% | 58.80 (46.3074.36) | 59.19 (45.70, 73.80) | 64.136 (52.000,75.674) | 0.143 |
| CD8+TeMRA+T% | 71.10 (47.8087.00) | 75.80 (47.60, 89.70) | 76.399 (48.151,88.800) | 0.709 |
| CD8+TN+T% | 28.06 (9.7563.50) | 30.30 (14.40, 67.10) | 29.100 (8.942,64.100) | 0.721 |
| MDSC | 1.89 (0.389.52) | 2.61 (0.30, 7.06) | 1.319 (0.416,6.030) | 0.711 |
| PMN_MDSC | 0.47 (0.005.11) | 0.64 (0.00, 5.47) | 0.698 (0.004,4.290) | 0.954 |
| M_MDSC | 0.10 (0.000.57) | 0.10 (0.00, 0.98) | 0.100 (0.000,0.400) | 0.568 |
| e_MDSC | 96.16 (90.8698.76) | 96.43 (91.93, 98.64) | 96.162 (90.864,98.600) | 0.726 |
| Th1 | 19.30 (11.4027.50) | 19.45 (12.25, 28.00) | 19.400 (12.100,28.672) | 0.817 |
| Th2 | 56.30 (41.0168.00) | 56.48 (39.30, 69.64) | 52.397 (38.222,66.000) | 0.485 |
| Th17 | 10.37 (5.7516.40) | 10.30 (6.36, 16.40) | 10.400 (6.760,16.700) | 0.760 |
| Treg | 8.08 (5.8811.10) | 8.17 (6.40, 11.49) | 8.080 (6.253,11.200) | 0.643 |
| CD4+CD45RA+T% | 16.40 (6.8127.82) | 14.20 (6.63, 28.55) | 16.246 (6.710,26.100) | 0.588 |
| CD4+CD45RO+T% | 61.40 (48.3074.50) | 61.16 (48.30, 74.10) | 61.163 (52.665,75.900) | 0.795 |
| CD8+CD45RA+T% | 31.50 (17.1048.30) | 39.10 (22.00, 49.66) | 31.100 (17.100,48.200) | 0.297 |
| CD8+CD45RA+T% | 37.50 (18.6053.21) | 33.10 (20.05, 53.16) | 40.200 (19.553,56.525) | 0.399 |
| CD4+CCR7+CD45+T% | 17.50 (10.4034.30) | 20.15 (10.76, 35.17) | 17.500 (7.826,34.139) | 0.732 |
| CD4+CCR7+CD45-T% | 54.10 (41.2068.48) | 52.75 (38.56, 68.14) | 57.100 (41.200,70.400) | 0.543 |
| CD4+CCR7-CD45+T% | 1.52 (0.423.92) | 1.74 (0.42, 3.91) | 1.860 (0.350,3.920) | 0.866 |
| CD4+CCR7-CD45-T% | 12.20 (3.8331.70) | 12.81 (3.71, 32.60) | 10.482 (2.510,31.700) | 0.544 |
| CD8+CCR7+CD45+T% | 28.40 (16.1044.80) | 28.30 (17.03, 44.47) | 28.300 (16.097,41.562) | 0.937 |
| CD8+CCR7+CD45-T% | 40.20 (22.7056.29) | 43.65 (20.32, 56.29) | 39.800 (19.622,52.900) | 0.852 |
| CD8+CCR7-CD45+T% | 11.00 (3.4526.00) | 8.95 (3.52, 21.62) | 9.411 (2.150,21.800) | 0.343 |
| CD8+CCR7-CD45-T% | 4.92 (1.8015.30) | 5.66 (1.74, 18.37) | 3.490 (1.330,15.300) | 0.241 |
