# Supplementary material for: Establishment and validation of the prediction model based on lymphocyte subsets for acute kidney injury in sepsis patients
Source: Front Immunol. 2025 Sep 25;16:1674673. doi: 10.3389/fimmu.2025.1674673 (PMC12507742; doi:10.3389/fimmu.2025.1674673)
Supplement: Supplementary file 6 [file Table6.docx]

**Table S6** Comparisons of the 3^rd^ lymphocyte subsets among training, validation and test sets

| **Variables** | **Training set** | **Validation set** | **Test set** | ***P*** |
| --- | --- | --- | --- | --- |
| nCD64 index | 5.04 (1.48, 13.14) | 4.49 (1.41, 12.23) | 6.20 (1.89,21.09) | 0.387 |
| CD3+T% | 62.70 (55.31, 74.14) | 63.79 (55.40, 74.63) | 63.55 (56.03,74.14) | 0.605 |
| CD4+T% | 36.02 (27.16, 46.52) | 33.42 (24.19, 46.38) | 34.44 (26.44,46.00) | 0.793 |
| CD8+T% | 22.91 (17.02, 31.58) | 23.63 (16.85, 32.74) | 22.91 (16.98,32.78) | 0.962 |
| CD4+CD8+T% | 1.45 (0.86, 2.53) | 1.47 (0.88, 2.60) | 1.46 (0.84,2.48) | 0.961 |
| CD4-CD8-T% | 3.95 (1.88, 6.42) | 3.91 (1.95, 6.31) | 3.98 (1.81,8.00) | 0.712 |
| CD16+CD56+NK% | 9.62 (5.38, 17.86) | 9.14 (5.68, 18.68) | 9.14 (4.55,18.73) | 0.825 |
| CD19+B% | 18.48 (11.02, 27.20) | 18.96 (11.16, 25.94) | 18.08 (7.92,27.20) | 0.921 |
| NKT% | 3.84 (2.46, 8.28) | 3.92 (2.56, 8.28) | 3.84 (2.08,7.69) | 0.464 |
| CD3+T count | 525.00 (254.00, 1050.00) | 503.47 (240.00, 1020.04) | 526.00 (254.00,1201.00) | 0.790 |
| CD4+T count | 288.00 (146.00, 640.33) | 288.00 (147.50, 596.63) | 320.00 (149.00,663.00) | 0.779 |
| CD8+T count | 177.00 (64.00, 336.00) | 176.00 (55.00, 370.00) | 180.00 (69.00,381.00) | 0.830 |
| CD4/CD8 | 1.63 (0.69, 2.38) | 1.45 (0.66, 2.42) | 1.75 (0.65,2.38) | 0.946 |
| CD4+CD8+T count | 7.00 (2.00, 15.94) | 7.50 (2.00, 16.88) | 7.00 (2.00,20.00) | 0.730 |
| CD4-CD8-T count | 15.00 (7.00, 39.94) | 15.50 (8.00, 40.00) | 18.00 (5.15,51.00) | 0.505 |
| CD16+CD56+T count | 92.00 (41.00, 128.00) | 105.00 (38.00, 159.25) | 94.00 (33.00,125.00) | 0.617 |
| CD19+B count | 143.00 (61.00, 230.00) | 114.00 (52.00, 195.26) | 143.00 (62.00,267.00) | 0.182 |
| lymphocyte count | 797.00 (418.00, 1534.00) | 684.62 (382.00, 1534.00) | 833.50 (404.00,1850.00) | 0.894 |
| NKT count | 39.00 (12.00, 73.00) | 43.50 (10.00, 76.00) | 37.00 (12.00,70.00) | 0.971 |
| CD4+CD28+T% | 88.36 (67.70, 96.90) | 88.58 (71.65, 96.72) | 85.80 (62.30,96.20) | 0.346 |
| CD4+CD38+T% | 48.10 (20.60, 68.66) | 42.20 (12.16, 65.44) | 44.40 (15.24,66.00) | 0.192 |
| CD4+CD69+T% | 65.36 (39.30, 83.20) | 60.13 (38.00, 83.00) | 68.70 (39.30,83.20) | 0.698 |
| CD8+CD28+T% | 41.80 (30.60, 63.83) | 42.49 (30.80, 63.83) | 41.50 (29.80,64.20) | 0.926 |
| CD8+CD38+T% | 41.98 (19.00, 64.96) | 34.48 (10.00, 64.22) | 37.34 (14.17,62.00) | 0.149 |
| CD8+CD69+T% | 38.30 (24.50, 54.40) | 36.06 (23.70, 53.01) | 39.18 (26.00,58.10) | 0.379 |
| CD155+T% | 45.38 (36.96, 56.97) | 46.50 (36.60, 58.23) | 41.43 (35.01,52.75) | 0.172 |
| CD4+BTLA+T% | 29.10 (17.60, 62.51) | 29.10 (19.20, 65.90) | 27.00 (17.00,54.40) | 0.629 |
| CD4+CTLA4+T% | 17.22 (6.37, 37.27) | 17.22 (8.34, 40.90) | 17.22 (8.34,42.59) | 0.581 |
| CD4+HLADR+T% | 62.10 (25.38, 95.27) | 62.00 (25.38, 95.23) | 57.10 (23.30,91.00) | 0.710 |
| CD4+LAG3+T% | 37.96 (27.17, 59.08) | 33.95 (23.50, 52.83) | 43.70 (27.40,64.00) | 0.073 |
| CD4+PD1+T% | 43.19 (30.12, 61.58) | 39.81 (29.20, 58.78) | 41.52 (32.90,61.58) | 0.447 |
| CD4+TIGIT+T% | 53.06 (28.20, 76.74) | 49.30 (28.20, 77.30) | 61.55 (32.46,77.30) | 0.266 |
| CD4+TIM3+T% | 31.40 (13.83, 46.61) | 27.64 (13.30, 46.74) | 28.66 (13.93,48.14) | 0.895 |
| CD4+TcM+T% | 84.38 (74.90, 91.60) | 84.40 (75.05, 91.90) | 83.10 (74.90,91.52) | 0.664 |
| CD4+TeM+T% | 57.02 (26.88, 82.20) | 62.10 (31.78, 82.90) | 48.03 (22.30,82.20) | 0.066 |
| CD4+TeMRA+T% | 19.42 (1.45, 55.10) | 18.61 (0.86, 63.30) | 20.90 (0.65,55.10) | 0.948 |
| CD4+TN+T% | 98.93 (96.14, 99.91) | 99.38 (96.73, 99.96) | 98.70 (95.50,99.95) | 0.637 |
| CD8+BTLA+T% | 36.23 (20.36, 52.18) | 36.48 (19.84, 52.13) | 35.90 (18.13,49.70) | 0.463 |
| CD8+CTLA4+T% | 20.00 (8.00, 30.10) | 20.46 (9.73, 30.27) | 20.12 (7.94,30.27) | 0.877 |
| CD8+HLADR+T% | 64.50 (34.08, 87.15) | 61.60 (32.40, 85.84) | 68.62 (43.10,89.10) | 0.341 |
| CD8+LAG3+T% | 27.60 (11.54, 40.20) | 27.95 (12.40, 38.71) | 28.10 (12.40,39.78) | 0.944 |
| CD8+PD1+T% | 22.20 (12.60, 30.20) | 20.00 (12.64, 28.75) | 23.90 (12.60,30.20) | 0.683 |
| CD8+TIGIT+T% | 54.40 (17.00, 80.69) | 61.10 (19.12, 83.27) | 55.61 (15.65,80.69) | 0.559 |
| CD8+TIM3+T% | 36.83 (23.40, 52.90) | 38.10 (21.10, 54.30) | 36.20 (18.61,48.50) | 0.365 |
| CD8+TcM+T% | 61.66 (30.42, 76.31) | 56.69 (28.80, 75.65) | 62.87 (34.10,77.80) | 0.458 |
| CD8+TeM+T% | 54.50 (39.70, 69.10) | 55.15 (42.09, 73.41) | 53.14 (36.00,68.21) | 0.315 |
| CD8+TeMRA+T% | 70.50 (47.80, 89.08) | 71.87 (47.80, 91.60) | 71.53 (51.61,90.40) | 0.485 |
| CD8+TN+T% | 48.60 (18.98, 75.48) | 46.02 (18.98, 74.69) | 54.52 (19.07,77.28) | 0.560 |
| MDSC | 2.35 (0.50, 12.38) | 2.35 (0.49, 7.74) | 2.43 (0.32,12.89) | 0.886 |
| PMN_MDSC | 0.05 (0.00, 0.82) | 0.04 (0.00, 0.97) | 0.01 (0.00,0.62) | 0.133 |
| M_MDSC | 0.06 (0.00, 1.33) | 0.18 (0.00, 2.38) | 0.02 (0.00,1.12) | 0.912 |
| e_MDSC | 98.60 (92.80, 99.80) | 98.29 (89.10, 99.80) | 99.00 (94.10,100.00) | 0.695 |
| Th1 | 24.90 (15.30, 36.80) | 24.95 (14.30, 37.73) | 26.70 (17.58,37.00) | 0.326 |
| Th2 | 54.30 (39.18, 65.60) | 55.05 (37.12, 68.37) | 51.93 (37.10,64.20) | 0.535 |
| Th17 | 8.48 (4.49, 12.40) | 9.00 (3.65, 14.12) | 9.21 (4.49,13.90) | 0.383 |
| Treg | 9.18 (7.35, 11.45) | 9.29 (7.32, 11.60) | 9.12 (7.83,11.20) | 0.880 |
| CD4+CD45RA+T% | 13.80 (3.54, 31.90) | 11.81 (2.57, 28.28) | 13.57 (1.78,28.60) | 0.228 |
| CD4+CD45RO+T% | 62.54 (42.50, 79.10) | 65.54 (42.50, 78.30) | 65.80 (48.78,79.10) | 0.457 |
| CD8+CD45RA+T% | 28.81 (13.10, 46.03) | 26.80 (12.20, 43.12) | 28.90 (17.22,45.27) | 0.686 |
| CD8+CD45RA+T% | 35.60 (22.80, 50.30) | 35.65 (21.66, 48.53) | 39.17 (28.89,54.49) | 0.079 |
| CD4+CCR7+CD45+T% | 21.44 (11.50, 38.80) | 22.09 (13.11, 42.05) | 18.88 (11.50,38.53) | 0.710 |
| CD4+CCR7+CD45-T% | 58.70 (45.66, 69.80) | 53.22 (42.09, 66.37) | 58.70 (43.30,70.00) | 0.064 |
| CD4+CCR7-CD45+T% | 1.19 (0.26, 3.03) | 1.11 (0.24, 2.79) | 1.04 (0.26,3.04) | 0.958 |
| CD4+CCR7-CD45-T% | 9.29 (2.52, 18.55) | 9.27 (3.61, 20.51) | 10.40 (2.46,26.85) | 0.737 |
| CD8+CCR7+CD45+T% | 22.70 (9.40, 42.80) | 22.70 (10.12, 40.79) | 15.49 (7.96,37.50) | 0.054 |
| CD8+CCR7+CD45-T% | 38.48 (21.88, 53.73) | 39.33 (24.92, 57.65) | 39.75 (23.00,51.20) | 0.717 |
| CD8+CCR7-CD45+T% | 10.50 (3.70, 26.60) | 11.10 (3.54, 25.35) | 9.23 (3.79,25.90) | 0.765 |
| CD8+CCR7-CD45-T% | 11.18 (2.31, 26.30) | 12.10 (2.03, 26.36) | 16.43 (2.52,25.40) | 0.739 |
